# Systematic Review of Safety and Efficacy of IL-1-Targeted Biologics in Treating Immune-Mediated Disorders

**DOI:** 10.3389/fimmu.2022.888392

**Published:** 2022-07-06

**Authors:** Dennis D. Arnold, Ayla Yalamanoglu, Onur Boyman

**Affiliations:** ^1^ Department of Immunology, University Hospital Zurich, Zurich, Switzerland; ^2^ Faculty of Medicine, University of Zurich, Zurich, Switzerland

**Keywords:** IL-1, anakinra, bermekimab, canakinumab, gevokizumab, rilonacept, autoinflammatory disease, immune-mediated

## Abstract

**Background:**

The cytokine interleukin (IL)-1 plays a pivotal role in immune-mediated disorders, particularly in autoinflammatory diseases. Targeting this cytokine proved to be efficacious in treating numerous IL-1-mediated pathologies. Currently, three IL-1 blockers are approved, namely anakinra, canakinumab and rilonacept, and two additional ones are expected to receive approval, namely gevokizumab and bermekimab. However, there is no systematic review on the safety and efficacy of these biologics in treating immune-mediated diseases.

**Objective:**

To evaluate safety and efficacy of anakinra, canakinumab, rilonacept, gevokizumab, and bermekimab for the treatment of immune-mediated disorders compared to placebo, standard-of-care treatment or other biologics.

**Methods:**

The PRISMA checklist guided the reporting of the data. We searched the PubMed database between 1 January 1984 and 31 December 2020 focusing on immune-mediated disorders. Our PubMed literature search identified 7363 articles. After screening titles and abstracts for the inclusion and exclusion criteria and assessing full texts, 75 articles were included in a narrative synthesis.

**Results:**

Anakinra was both efficacious and safe in treating cryopyrin-associated periodic syndromes (CAPS), familial Mediterranean fever (FMF), gout, macrophage activation syndrome, recurrent pericarditis, rheumatoid arthritis (RA), and systemic juvenile idiopathic arthritis (sJIA). Conversely, anakinra failed to show efficacy in graft-versus-host disease, Sjögren’s syndrome, and type 1 diabetes mellitus (T1DM). Canakinumab showed efficacy in treating CAPS, FMF, gout, hyper-IgD syndrome, RA, Schnitzler’s syndrome, sJIA, and TNF receptor-associated periodic syndrome. However, use of canakinumab in the treatment of adult-onset Still’s disease and T1DM revealed negative results. Rilonacept was efficacious and safe for the treatment of CAPS, FMF, recurrent pericarditis, and sJIA. Contrarily, Rilonacept did not reach superiority compared to placebo in the treatment of T1DM. Gevokizumab showed mixed results in treating Behçet’s disease-associated uveitis and no benefit when assessed in T1DM. Bermekimab achieved promising results in the treatment of hidradenitis suppurativa.

**Conclusions:**

This systematic review of IL-1-targeting biologics summarizes the current state of research, safety, and clinical efficacy of anakinra, bermekimab, canakinumab, gevokizumab, and rilonacept in treating immune-mediated disorders.

**Systematic Review Registration:**

https://www.crd.york.ac.uk/PROSPERO/, identifier CRD42021228547.

## Introduction

Immunity and immunotolerance employ both antigen-specific and antigen-independent mechanisms that need to be regulated to protect an individual from internal and external dangers. A correct functioning antigen-specific part, also known as adaptive immunity, is a well-balanced equilibrium of self-tolerance and detection of non-self. The actions of adaptive immunity rely on the complex interactions of T and B cells. A dysregulation of adaptive immune responses with recognition of self-antigens presented by major histocompatibility complex (MHC) molecules can result in prototypic systemic and organ-specific autoimmune diseases that feature auto-reactive T cells and autoantibodies, such as rheumatoid arthritis (RA), Sjögren’s syndrome (SjS), and systemic lupus erythematosus (SLE) (1). Contrarily, overactivation of innate immunity can also harm self-tissues and, thus, result in so-called autoinflammatory disease that does not feature typical auto-reactive T cells and autoantibodies and is independent of MHC molecules (2). Autoinflammatory disorders arise either by uncontrolled activation of proinflammatory components or by a lack of anti-inflammatory mechanisms, leading to dysregulated activation of innate immune cells, including typically neutrophil granulocytes, monocytes, and proinflammatory cytokines (3).

In the past decades, treatments for immune-mediated disorders consisted of glucocorticoids (GCs) and GC-sparing immunosuppressive drugs. However, these medications bare an increased risk for long-term adverse side effects, such as severe infections, skin cancer, and GC-induced trophic and functional impairment of certain tissues (4). Hence more precise biologic agents (also called biologics) were developed since the 1990s (5). These biologics bind to molecularly defined targets and, thus, they minimize adverse side and off-target effects.

The interleukin-1 (IL-1) molecules IL-1α and IL-1β are prototypic proinflammatory cytokines that act by binding to their common IL-1 receptor (IL-1R), made of IL-1R1 and IL-1R3 (also called IL-1R accessory protein), which initiates downstream signals culminating in inflammatory processes. IL-1R antagonist (IL-1Ra), a glycosylated natural antagonist, can bind to IL-1R1 and inhibit association of IL-1R1 with IL-1α and IL-1β. Biologics, including monoclonal antibodies (mAbs) and recombinant receptor proteins fused to human immunoglobulin G (IgG) fragments, targeting IL-1 and the IL-1R have benefitted the treatment of several immune-mediated disorders, notably, autoinflammatory diseases (6, 7).

The first approved IL-1-targeted therapy was anakinra, an aglycosylated recombinant IL-1Ra. Similar to natural endogenous IL-1Ra, anakinra binds to IL-1R1 and competitively prevents association of both IL-1α and IL-1β with IL-1R1 (3, 8). Subsequently, two additional drugs targeting the IL-1 pathway were approved, namely canakinumab and rilonacept. Canakinumab is a neutralizing, IgG1-type mAb directed to IL-1β that prevents binding of IL-1β to IL-1R1. Rilonacept consists of the extracellular domains of IL-1R1 and IL-1R3 that are fused to a fragment crystallizable (Fc) part of human IgG1; thus, rilonacept functions as a soluble decoy receptor for IL-1α and IL-1β. Furthermore, gevokizumab is a neutralizing humanized mAb specific to IL-1β (9). Bermekimab is a fully human mAb targeting and neutralizing IL-1α (10). The present study was conducted to provide a systematic review on the safety and efficacy of these IL-1-targeting biologics in treating immune-mediated diseases.

## Methods

### Study Design and Protocol Registration

This systematic review was guided by the PRISMA (**Table 1**) checklist. The PROSPERO number registered for our protocol was CRD42021228547.

**Table 1:** The Preferred Reporting of Systematic Reviews and Meta-Analyses (PRISMA) checklist.

Section and Topic	Item #	Checklist item	Reported on page #
**TITLE**			
Title	1	Identify the report as a systematic review.	1
**ABSTRACT**			
Abstract	2	See the PRISMA 2020 for Abstracts checklist.	2
**INTRODUCTION**			
Rationale	3	Describe the rationale for the review in the context of existing knowledge.	2
Objectives	4	Provide an explicit statement of the objective(s) or question(s) the review addresses.	2, 4
**METHODS**			
Eligibility criteria	5	Specify the inclusion and exclusion criteria for the review and how studies were grouped for the syntheses.	2
Information sources	6	Specify all databases, registers, websites, organisations, reference lists and other sources searched or consulted to identify studies. Specify the date when each source was last searched or consulted.	2
Search strategy	7	Present the full search strategies for all databases, registers and websites, including any filters and limits used.	2, **Table S1**
Selection process	8	Specify the methods used to decide whether a study met the inclusion criteria of the review, including how many reviewers screened each record and each report retrieved, whether they worked independently, and if applicable, details of automation tools used in the process.	2
Data collection process	9	Specify the methods used to collect data from reports, including how many reviewers collected data from each report, whether they worked independently, any processes for obtaining or confirming data from study investigators, and if applicable, details of automation tools used in the process.	4
Data items	10a	List and define all outcomes for which data were sought. Specify whether all results that were compatible with each outcome domain in each study were sought (e.g. for all measures, time points, analyses), and if not, the methods used to decide which results to collect.	2
	10b	List and define all other variables for which data were sought (e.g. participant and intervention characteristics, funding sources). Describe any assumptions made about any missing or unclear information.	2
Study risk of bias assessment	11	Specify the methods used to assess risk of bias in the included studies, including details of the tool(s) used, how many reviewers assessed each study and whether they worked independently, and if applicable, details of automation tools used in the process.	4, **Table S2**
Effect measures	12	Specify for each outcome the effect measure(s) (e.g. risk ratio, mean difference) used in the synthesis or presentation of results.	2, 4
Synthesis methods	13a	Describe the processes used to decide which studies were eligible for each synthesis (e.g. tabulating the study intervention characteristics and comparing against the planned groups for each synthesis (item #5)).	NA
	13b	Describe any methods required to prepare the data for presentation or synthesis, such as handling of missing summary statistics, or data conversions.	NA
	13c	Describe any methods used to tabulate or visually display results of individual studies and syntheses.	NA
	13d	Describe any methods used to synthesize results and provide a rationale for the choice(s). If meta-analysis was performed, describe the model(s), method(s) to identify the presence and extent of statistical heterogeneity, and software package(s) used.	NA
	13e	Describe any methods used to explore possible causes of heterogeneity among study results (e.g. subgroup analysis, meta-regression).	NA
	13f	Describe any sensitivity analyses conducted to assess robustness of the synthesized results.	NA
Reporting bias assessment	14	Describe any methods used to assess risk of bias due to missing results in a synthesis (arising from reporting biases).	4
Certainty assessment	15	Describe any methods used to assess certainty (or confidence) in the body of evidence for an outcome.	NA
**RESULTS**			
Study selection	16a	Describe the results of the search and selection process, from the number of records identified in the search to the number of studies included in the review, ideally using a flow diagram.	4, **Figure 1**, **Table S3**
	16b	Cite studies that might appear to meet the inclusion criteria, but which were excluded, and explain why they were excluded.	4, **Table S3**
Study characteristics	17	Cite each included study and present its characteristics.	4, **Table S4**
Risk of bias in studies	18	Present assessments of risk of bias for each included study.	14, **Table S5**
Results of individual studies	19	For all outcomes, present, for each study: (a) summary statistics for each group (where appropriate) and (b) an effect estimate and its precision (e.g. confidence/credible interval), ideally using structured tables or plots.	4-14
Results of syntheses	20a	For each synthesis, briefly summarise the characteristics and risk of bias among contributing studies.	14, **Table S5**
	20b	Present results of all statistical syntheses conducted. If meta-analysis was done, present for each the summary estimate and its precision (e.g. confidence/credible interval) and measures of statistical heterogeneity. If comparing groups, describe the direction of the effect.	NA
	20c	Present results of all investigations of possible causes of heterogeneity among study results.	NA
	20d	Present results of all sensitivity analyses conducted to assess the robustness of the synthesized results.	NA
Reporting biases	21	Present assessments of risk of bias due to missing results (arising from reporting biases) for each synthesis assessed.	14, **Table S5**
Certainty of evidence	22	Present assessments of certainty (or confidence) in the body of evidence for each outcome assessed.	NA
**DISCUSSION**			
Discussion	23a	Provide a general interpretation of the results in the context of other evidence.	14, 15, 18
	23b	Discuss any limitations of the evidence included in the review.	15, 18
	23c	Discuss any limitations of the review processes used.	15, 18
	23d	Discuss implications of the results for practice, policy, and future research.	18
**OTHER INFORMATION**			
Registration and protocol	24a	Provide registration information for the review, including register name and registration number, or state that the review was not registered.	2
	24b	Indicate where the review protocol can be accessed, or state that a protocol was not prepared.	18
	24c	Describe and explain any amendments to information provided at registration or in the protocol.	NA
Support	25	Describe sources of financial or non-financial support for the review, and the role of the funders or sponsors in the review.	18
Competing interests	26	Declare any competing interests of review authors.	18
Availability of data, code and other materials	27	Report which of the following are publicly available and where they can be found: template data collection forms; data extracted from included studies; data used for all analyses; analytic code; any other materials used in the review.	18

NA, not applicable.

### Search Strategy

We searched the PubMed database between 1 January 1984 and 31 December 2020. We defined the full search strategy and all search terms in advance. Specific search terms and keywords are provided in **Table S1**. We excluded publications not concerning immune-mediated disorders. Secondly, we used automation tools such as PubMed filters to exclude works not fulfilling our inclusion criteria. Subsequently, we screened the papers for title, abstract and content. If publications were not available through open or institutional access, we contacted the study authors.

### Eligibility Criteria

As established in our previous studies (11–14), we included randomized controlled trials (RCTs), their extension trials and their substudies with predefined endpoints. If there were no RCTs, we included prospective case series including at least three patients and non-randomized clinical studies with at least five patients per intervention group. We excluded retrospective trials, post-hoc analyses, meta-analyses, reviews, studies made from registries and studies carried out on animal models or where the primary endpoint was non-clinical. Studies had to be available in English or German.

### Study Selection, Data Collection Process and Analysis

Two authors (DA and OB) developed and tested a data extraction sheet, whereupon two authors independently (DA and AY) searched PubMed according to the predefined search terms, checked titles and abstracts, carried out a full-text review of the selected studies, and extracted the relevant data. Any disagreements about study inclusion were resolved by consensus.

### Risk of Bias Assessment

DA used a modified version of the Downs and Black tool (see **Table S2**) to assess the selected studies for bias (15). A scoring sheet was used to rank for the risk of bias, the more points, the lower the risk of bias. We categorized in low (23-28 points), medium (15-22 points) and high (0-14 points) risk. There was a maximum of 28 points in 4 categories: (i) reporting, (ii) external validity, (iii) internal validity, and (iv) power.

As we limited our research strategy to the PubMed database, the reference list of these studies, and the expertise of the authors involved, we did not conduct a risk-of-bias assessment across the studies, as we believed the risk of publication bias was high.

### Principal Summary Measures and Synthesis of Results

The aim of this systematic review was to provide a structured and complete overview of the current available studies assessing safety and efficacy of anakinra, bermekimab, canakinumab, gevokizumab, and rilonacept as well as their influence on quality of life (QoL) when used in immune-mediated diseases. Since we wanted to give an overview, including also rare diseases, we did not specify in more detail these endpoints in order not to exclude potentially important studies.

## Results

### Study Selection and Characteristics

**Figure 1:**
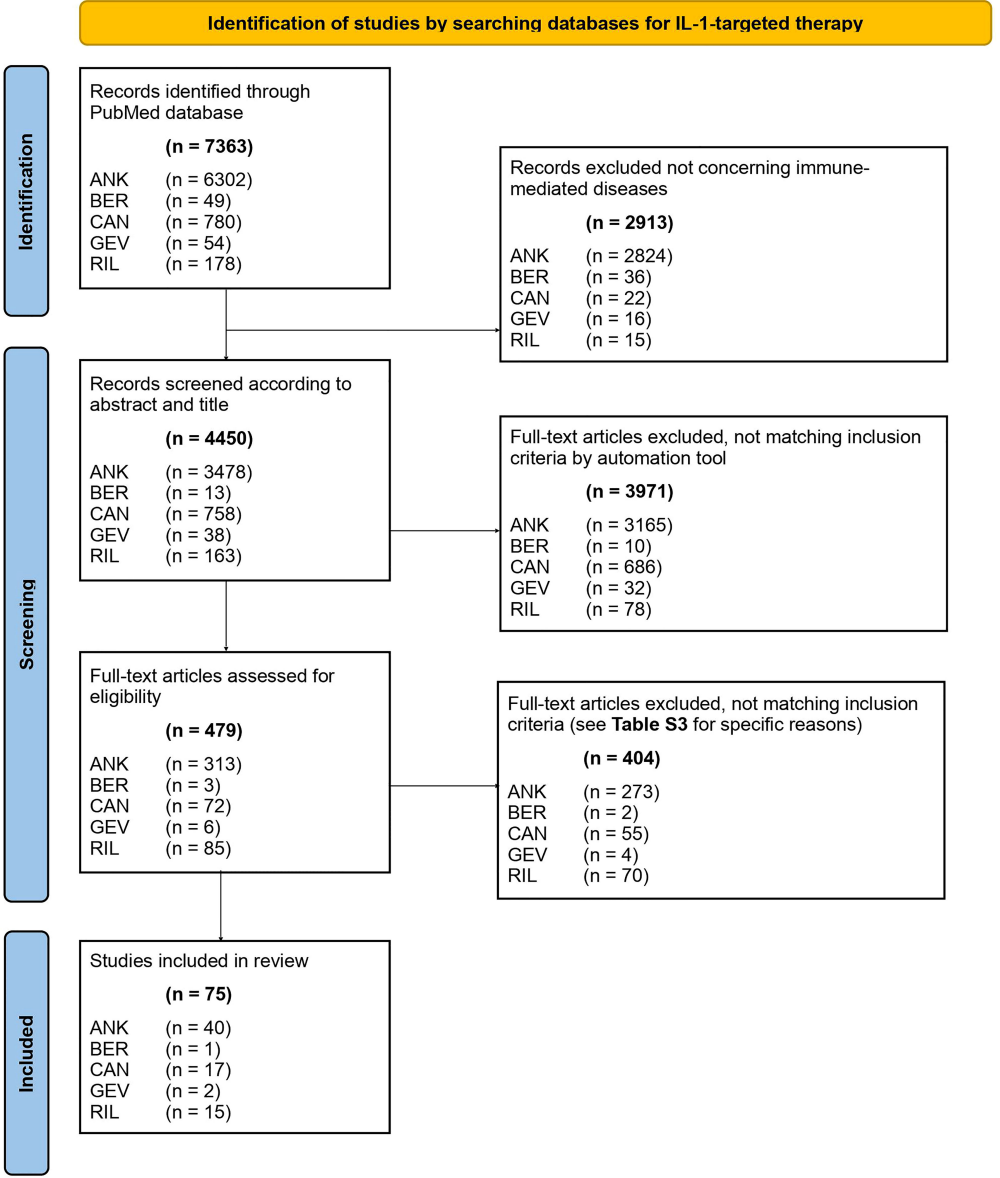
PRISMA 2020 flow diagram of literature search. ANK, anakinra; BER, bermekimab; CAN, canakinumab; GEV, gevokizumab; RIL, rilonacept.

Our PubMed search resulted in 7363 articles. We screened 479 of them for title and abstract and finally included 75 publications in our systematic review (**Figure 1**), by using the PRISMA checklist (16). The main exclusion characteristics are available in **Table S3**. Characteristics of all studies included are available in **Table S4**. We decided to exclude the work of Bottin et al. (17) treating refractory scleritis by use of anakinra because of the heterogeneity of the underlying systemic conditions that included both patients with autoinflammatory and autoimmune diseases. Furthermore, we did not find studies matching our inclusion criteria for the use of IL-1-targeted biologics in the treatment of pyogenic arthritis, pyoderma gangrenosum and acne (also termed PAPA), periodic fever, aphthous stomatitis, pharyngitis, and adenitis (also known as PFAPA), and relapsing polychondritis; thus, these three immune-mediated diseases are not discussed below.

### Synthesized Findings

This chapter summarizes the most important findings. The biologics and diseases are discussed in alphabetical order.

#### Adult-Onset Still’s Disease

##### Anakinra

Only one open-label, randomized, multicenter trial met our criteria, which tested disease remission according to eight specific measures with use of anakinra within two parallel groups of adult-onset Still’s disease (AOSD) patients (18). 22 patients were randomized to either receiving anakinra or standard of care with disease-modifying anti-rheumatic drugs (DMARDs), such as methotrexate, azathioprine, leflunomide, cyclosporine A or sulphasalazine, plus GCs. 58% of patients in the anakinra group compared to 50% in the DMARD group showed complete remission. This difference did not reach statistical significance. Adverse events (AEs) were not significantly different between the study groups, and there were no reported severe adverse events (SAEs). Secondary endpoints or QoL were not investigated.

##### Canakinumab

We found one double-blind RCT (the CONSIDER trial) assessing treatment with canakinumab in 36 patients with AOSD (19). This trial investigated the difference in the 28-joint disease activity score (DAS28) at week 12. The investigators found that 66% in the canakinumab group compared to 41% with placebo reached the primary endpoint, although this difference was not statistically significant. Several secondary endpoints assessing disease activity, fever episodes, health assessment questionnaire (HAQ), American College of Rheumatology (ACR) and European League Against Rheumatism (EULAR) responses showed larger improvements in the canakinumab group than in placebo; however, these differences were also not statistically significant.

During the first 12 weeks, 47 AEs were observed in patients receiving canakinumab compared to 21 AEs in placebo. In the 24-week follow-up period, where non-responders from the placebo group were given the possibility to switch to the canakinumab group, nine SAEs were reported, including four in the canakinumab group and five in placebo. Consequently, the exposure time was approximately three times higher in the canakinumab group. However, AE rates per 100 patient-years were equal in both groups. Furthermore, there was a significant change in physical QoL, measured with the disability index QoL 36-item short form (SF-36), but not in mental QoL, in the canakinumab group with an improvement of 10 points compared to no change in placebo.

##### Rilonacept

One case series with three patients fulfilled our inclusion criteria (20). Three patients suffering from AOSD were treated with rilonacept. In all three cases a clinical and laboratory response was evident. No AEs or SAEs were reported. There was no information on QoL.

*Synopsis:* Evidence on their use in AOSD showed no advantage of anakinra, mixed results for canakinumab, and positive effects with rilonacept in a small case series. Larger and placebo-controlled RCTs are necessary to assess whether IL-1-targeting biologics are beneficial in AOSD.

#### Behçet’s Disease

##### Anakinra

We found one open-label design study with six patients and one case series with nine patients investigating treatment responses of anakinra in Behçet’s disease (BD) patients (21, 22). In the open-label study, 33% of patients showed a complete response defined by the absence of oral and genital ulcers on two consecutive monthly visits. In the case series, 77% of patients showed an initial treatment response, although 88% showed an occurrence of relapse during the 19-month follow-up. The open-label study could not demonstrate a significant difference in QoL. Both studies reported in total 38 AEs, of which four were categorized as SAEs, during the 12-19-month follow-up period (21, 22).

##### Canakinumab

Vitale and colleagues described a case report of three patients with BD treated with canakinumab (23). All three patients showed a complete clinical response. These patients were followed up for 6-12 months and no AEs or SAEs were reported. QoL was not investigated.

##### Gevokizumab

We found one study, fulfilling our inclusion criteria, that investigated gevokizumab in treating BD-associated uveitis (24). This phase-two RCT included 83 patients receiving either gevokizumab (40 patients) or placebo (43 patients). Compared to placebo, gevokizumab did not significantly affect the time to first ocular exacerbation. However, the investigators reported a significant reduction of GCs to less than 10 mg prednisone at disease recurrence, with 92% of patients achieving this GC dose in the gevokizumab arm compared to 80% in the placebo arm. There was no significant difference with gevokizumab compared to placebo in the incidence of AEs (92.7% and 93%, respectively) and SAEs (13 and 14 events, respectively) throughout the 27-month study period. QoL was not investigated.

*Synopsis:* In mucocutaneous BD, anakinra and canakinumab showed promising results. In BD-associated uveitis, gevokizumab failed to show superiority over placebo in terms of prevention of exacerbation, but patients receiving gevokizumab were more likely to reduce their GC dose. Use of IL-1-targeting biologics in BD needs further investigation in larger studies.

#### Cryopyrin-Associated Periodic Syndromes

##### Anakinra

Four eligible studies met the inclusion criteria, but none of them were RCTs. These included three open-label studies with total 89 patients with cryopyrin-associated periodic syndromes (CAPS) and one case series with three individuals. They investigated clinical and laboratory response, QoL and AEs (25–28). Sibley et al. found a significant response at six months in all 26 patients treated with anakinra (28). After 12 and 60 months of treatment with anakinra, clinical and laboratory systemic remission was achieved in 46% and 65% of patients, respectively (28). Eskola et al. found a clinical and laboratory complete response in one patient (33%) and partial response in the other two patients (67%) (27). QoL was the primary endpoint in the study of Lepore et al. (26); the authors found that introduction of treatment was associated with a notable and sustained improvement of QoL. During the five-year follow-up period of the Kullenberg et al. study, 1233 AEs were reported, resulting overall in 7.7 events per patient-year and occurring in 95% of all treated patients (25). 24 cases were categorized as SAEs. The most common SAEs were infections.

We found two single-center, open-label, prospective observational studies comparing safety and efficacy of anakinra in patients suffering from MWS, which is a disease belonging to the CAPS (29, 30). A first study of Kuemmerle et al. in 12 patients showed a 100% treatment response to anakinra at week two, measured by improvement of disease activity score (DAS) (29). Markers of inflammation improved in all but one patient. 14 AEs were observed, but no SAEs were seen in the 22-114 months of follow-up. In a second study of Kuemmerle et al. comparing anakinra to canakinumab in 12 patients each, both treatments showed a significant reduction of DAS, which was comparable in both treatment arms (30). 75% showed persistent disease remission in the anakinra group, compared to 93% in the canakinumab group at the long-term study end-point, and significant reductions of acute-phase reactants were observed in both treatment arms. This study reported 15 AEs – nine in anakinra and six in canakinumab – and one SAE in the canakinumab group, which was not considered to be associated with canakinumab. This second study by Kuemmerle et al. also showed a significant and sustained positive impact on QoL, as documented by all patient-derived measures (30).

##### Canakinumab

Two publications met our inclusion criteria. Both studies stemmed from the same RCT treating 31 patients with CAPS, 94% of whom suffered from MWS, with either canakinumab or placebo (31, 32). The primary endpoint of the study by Lachmann et al. was the proportion of patients with a relapse after study drug withdrawal (31). They showed that zero patients in the canakinumab group and 13 (81%) in the placebo group experienced a relapse. Furthermore, mean C-reactive protein (CRP) and serum amyloid A (SAA), as well as patient global assessment (PGA) were significantly lower in patients receiving canakinumab compared to placebo. 15 AEs were reported in the canakinumab group and 14 in placebo. Two SAEs occurred.

Koné-Paut et al. investigated different primary and secondary endpoints (32). They investigated the number of patients with a complete response at week eight, i.e. before blinding occurred, and found that 80% of patients receiving canakinumab responded. Moreover, maintenance of complete response at week 24 was 85% for the canakinumab group and 25% in placebo. Also, the investigators observed a positive change in QoL in all patients receiving canakinumab during the eight-week phase before blinding, with QoL reaching levels of the general population.

##### Rilonacept

Two consecutive RCTs evaluated the effects of rilonacept in CAPS (33). Trial number one (T1) investigated the efficacy under treatment with rilonacept, whereas the second trial (T2) assessed the maintenance of improvements achieved in T1. T1 included a total of 47 patients, of which 23 were treated with rilonacept and 24 with placebo. There was a mean reduction of 84% in the key symptom score in the rilonacept group compared to 13% in placebo. More specifically, subjects receiving rilonacept experienced a reduction of at least 30% of all symptoms in 96%, a 50% reduction in 87%, and a 75% reduction in 70%. In the placebo group, these measures were significantly lower and amounted to 29%, 8%, and 0%, respectively.

In T2, 45 patients were treated with rilonacept for nine weeks. After this period of nine weeks, rilonacept was either changed to same dose of rilonacept in 22 patients or placebo in 23 patients. The rilonacept–rilonacept group maintained the achieved reduction in key symptoms, in number of multi-symptom days, in maximum severity of any symptoms, in mean change in GPA, and in laboratory markers of inflammation – i.e. CRP and SAA – compared to the rilonacept–placebo arm.

In T1, 74% of patients experienced AEs with rilonacept and 54% with placebo. The most frequent AEs were injection site reactions, which were reported with a rate that was three-fold higher than that with placebo treatment. Similar results were found in T2 where 68% of patients experienced AEs with rilonacept and 57% with placebo. No SAEs were reported in either of the two studies. QoL was not investigated.

*Synopsis:* On the basis of convincing safety and efficacy data, anakinra and canakinumab were approved by the European Medicines Agency (EMA) and the U.S. Food and Drug Administration (FDA) for the treatment of CAPS. Likewise, the EMA and FDA approved rilonacept for treating CAPS, however, the marketing-authorization holder of rilonacept withdrew its approval for commercial reasons.

#### Deficiency in IL-1 Receptor Antagonist

##### Rilonacept

One open-label study investigated treatment efficacy of rilonacept in six patients suffering from deficiency in IL-1 receptor antagonist (DIRA) (34). Achievement of remission or maintenance of remission (if patients were already receiving IL-1-blocking therapy before the initiation of rilonacept) was seen in all patients, although five of six patients required dose escalation to achieve this effect. Furthermore, improvements in patient’s questionnaires, diary scores, acute phase reactants, growth, and weight were reported. The investigators also observed normalization of bone minerality. All six patients experienced AEs, but none of the subjects reported SAEs. Improvement of QoL was reported by all patients.

*Synopsis:* This small open-label study in six DIRA patients showed promising results. Thus, further investigations are warranted to assess efficacy and safety of rilonacept in treating DIRA.

#### Familial Mediterranean Fever

##### Anakinra

Only one RCT fulfilled our inclusion criteria assessing 25 patients with genetically confirmed familial Mediterranean fever (FMF) (35). Patients were randomized to receive placebo or anakinra, and in both cases concomitant colchicine and analgetics. Compared to placebo, anakinra showed a significant reduction of total number of attacks by 60% over the 16-week study period. Furthermore, there was a significant reduction in number of attacks per site, acute phase reactants, such as CRP and SAA, and a significant improvement of QoL by 50%. There were no significant differences in the number of AEs in both study groups and there was no reporting of SAEs.

##### Canakinumab

We found one RCT (the CLUSTER trial) and its extension trial fulfilling our inclusion criteria that assessed canakinumab treatment in 63 patients with FMF (36, 37). The primary endpoint of the RCT was clinical response (37). The investigators treated patients with either canakinumab or placebo and showed a complete response in 61% in the canakinumab group compared to 6% in the placebo arm. This difference was significant. Regarding secondary outcomes, 65% of patients in the canakinumab group had a PGA score less than 2 compared to 9% assigned to placebo. The acute phase reactants CRP and SAA were also significantly lower in the canakinumab group. The proportion of patients without a flare after eight weeks of treatment was 77.8% in canakinumab and 30% in placebo. All secondary endpoints were significant.

The CLUSTER extension trial investigated the long-term efficacy of canakinumab after open-label treatment with two different cumulative doses (either less or more than 2700 mg) of canakinumab in all patients, including the group previously treated with placebo (36). The investigators showed that 58% of patients had no flares during the 72-week follow-up period and 38% had only a single flare during this time. Incidence of flares were similar in both arms. A low PGA score could be maintained throughout the follow-up period without differences in both dose arms. Moreover, 90% of patients showed minimal disease activity at study end. Inflammatory markers remained low throughout the study.

Based on the RCT, the number of AEs was significantly higher in patients treated with canakinumab (16 per 100 patient-years) compared to placebo (8 per 100 patient-years). As for SAEs, three SAEs occurred in the canakinumab group compared to seven in placebo, which was interpreted as caused by a higher rate of FMF flares in the placebo group. QoL was not investigated. In the extension trial, 1.53 AEs (485 cases in total) and 0.08 SAEs (23 cases in total) per 100 patient-days were reported. The rate of AEs was higher in the group receiving a higher dose of canakinumab.

##### Rilonacept

One RCT investigated over one year the treatment efficacy and safety of rilonacept in 11 patients suffering from FMF (38). All included patients received one out of four different treatment regimens that included two three-month treatment courses with rilonacept and placebo in different sequences. A reduction by at least 40% of frequency of disease-specific flares was achieved by 76% of patients in the rilonacept treatment phase compared 39% of patients during the placebo phase. Furthermore, seven patients receiving rilonacept compared to zero treated with placebo remained relapse-free during treatment. Patients treated with rilonacept had a shorter period of attack duration (2.8 days) compared to placebo (3.2 days).

Significantly more AEs were recorded in the rilonacept arm (73 AEs) compared to the placebo arm (36 AEs). However, there was no significant difference in SAEs between rilonacept (4 SAEs) and placebo (3 SAEs). There was a statistically significant difference between treatment groups in the physical, but not in the psychological, aspects of QoL.

*Synopsis:*Based on convincing efficacy and safety data, anakinra was approved by the EMA and canakinumab by both the EMA and FDA for the treatment of FMF. Rilonacept appeared to be a promising treatment for FMF, and larger studies are needed to confirm the findings.

#### Gout

##### Anakinra

We found one RCT investigating 88 patients with a history of recurrent gout disease flares that were randomized to receive either anakinra or placebo once daily for five consecutive days (39). Colchicine, naproxen or prednisone (PDN) was allowed throughout the study. Treatment with anakinra was non-inferior to standard-of-care treatment, although there was no significant reduction in the primary endpoint, which consisted of a patient-reported rating scale. Nevertheless, pain relieve with anakinra was greater compared to placebo. There were no differences in the number of AEs and no SAEs were reported.

##### Canakinumab

Four RCTs met our inclusion criteria. Three studies investigated canakinumab as treatment of acute gout flares (40–42), whereas another study assessed canakinumab as a preventive treatment in acute gouty arthritis flares during initiation of allopurinol treatment (43). In the latter study, 391 individuals with acute gouty arthritis flares received either monthly canakinumab (283 patients) or daily colchicine (108 patients), which demonstrated that canakinumab reduced the mean number of gout flares per patient by 62-72% over the reduction observed in colchicine-treated individuals (43). The percentage of patients experiencing at least one flare was 15-27% in canakinumab versus 44% in colchicine and statistically significant. Furthermore, there was a 64-72% reduction in the risk of experiencing a flare under canakinumab versus colchicine after 16 weeks of treatment. Compared to colchicine, treatment with canakinumab also reduced the duration of flares, and CRP values remained consistently lower than with colchicine.

Three studies compared canakinumab to triamcinolone acetonide intramuscularly as an acute treatment of gout flares and found that canakinumab achieved in a higher number of patients no or mild pain compared to triamcinolone acetonide (41). Inflammatory markers, such as CRP and SAA, were normalized after seven days in most patients receiving canakinumab but remained elevated in triamcinolone acetonide (40). QoL was significantly better in the canakinumab arm compared to triamcinolone acetonide (41).

Overall, AEs were reported more frequently with use of canakinumab in 41-66% of cases in all four studies, compared with colchicine in 42-52% of individuals. However, one study reported a similar incidence of AEs across treatment groups, i.e. 54% with canakinumab versus 53% with colchicine, during a 24-week follow-up (43). SAEs occurred in 2.8-7.6% with canakinumab and 1.1-4.4% with triamcinolone acetonide (40–42), and in four cases with canakinumab versus one in colchicine (43).

##### Rilonacept

We found five RCTs that investigated rilonacept in the treatment of gout, with four (44–47) assessing the prevention of gout flares and one (48) the treatment of acute gout attacks. The latter study included 225 patients with an acute gout attack, treated with rilonacept with or without indomethacin (149 patients) or receiving placebo with indomethacin (collectively termed control group and consisting of 76 patients) (48). The primary endpoint was reduction in pain in the index joint, and this endpoint was not significantly different in the rilonacept group compared to the control group (reduction of 1.6 and 1.4, respectively). Also the secondary endpoints (mean change in pain at 24, 48, and 72 hours) revealed no significant differences when comparing rilonacept plus indomethacin to indomethacin alone. Furthermore, indomethacin alone was significantly superior to rilonacept monotherapy at all time points in terms of secondary endpoints. There was a significant CRP reduction in both study arms (rilonacept with or without indomethacin). Rescue therapy, defined as indomethacin 50 mg three times daily for one day, was needed in 3% of patients on rilonacept compared to 4.3% in the control group.

Three RCTs assessed the effect of rilonacept for the prevention of gout flares in a study population of 248 gout patients, treated with either two different doses of rilonacept (80 mg or 160 mg weekly; in 166 patients) or placebo (in 82 patients) (44–46). During the study period of 16 weeks, a significantly lower number of gout flares per patient were noted with 80 mg rilonacept (29 gout flares) and 160 mg rilonacept (28 gout flares) compared to 101 flares in the control group. This effect amounted to a 72% reduction compared to baseline in the rilonacept group and 0% in the placebo group. Furthermore, the duration of flares was significantly reduced with a mean of 3.0 days in both rilonacept groups compared to 11.2 days in the control arm. 25% of patients showed at least one gout flare during the study period in the rilonacept arm compared to 56% of patients in the placebo group.

Considering all RCTs, the frequency of AEs and SAEs was similar for rilonacept and placebo. Thus, in the above-mentioned three RCTs (44–46), AEs were observed in 65% of patients receiving rilonacept and in 61% treated with placebo. Eight SAEs were reported in the rilonacept and four in the placebo arm; hence, SAEs occurred with a similar frequency with rilonacept and placebo and none of the SAEs were considered related to the study drug.

Furthermore, a separate RCT (the RESURGE trial) investigated the safety of rilonacept when compared to placebo in the prevention of gout flares in 1315 patients (47). 824 patients were treated with rilonacept compared to 276 with placebo. The frequency of AEs and SAEs were comparable in both study arms, with 656 AEs (66%) occurring in the rilonacept group compared to 195 AEs (59%) in the placebo arm. Patients in the rilonacept group reported 31 SAEs (3.1%) compared to 13 SAEs (3.9%) in subjects receiving placebo. QoL was not investigated.

*Synopsis:* Safety and efficacy data demonstrated that anakinra and canakinumab were non-inferior to standard-of-care treatment in the treatment of acute gout flares. Based on these data, the EMA approved canakinumab for the treatment of gout. Rilonacept showed promising results in the prevention of gout flares but not in treating acute gout attacks.

#### Graft-Versus-Host Disease

##### Anakinra

One double-blind, stratified RCT investigated the efficacy of anakinra in preventing graft-versus-host disease (GvHD) after allogeneic bone marrow transplantation in two groups given either intravenous anakinra or placebo from day -4 through day 10, and both receiving a conditioning therapy consisting of cyclophosphamide, total body irradiation, cyclosporine A, and methotrexate (49). There was no difference in preventing grade B-D acute GvHD within the 100-day follow-up period. The overall survival, frequency of complications, and time to engraftment until 100 days after transplantation did not show any differences in both study groups. The study did not mention or analyze AEs or SAEs. QoL was not investigated either.

*Synopsis:* Anakinra did not show superiority over placebo in preventing GvHD after allogeneic bone marrow transplantation in the reported RCT. Thus, anakinra is not recommended for use in this indication.

#### Hidradenitis Suppurativa

##### Anakinra

A double-blind RCT, investigating the efficacy of anakinra in treating 20 hidradenitis suppurativa (HS) patients, met our inclusion criteria (50). 78% of the patients in the anakinra arm showed an improvement of DAS compared to only 20% in the placebo group. Furthermore, the time to a new exacerbation was significantly longer in the anakinra group compared to placebo. In total four AEs – three in the anakinra group and one in the placebo group – and no SAEs were reported. There was no difference in QoL between study arms.

##### Bermekimab

One RCT assessing bermekimab versus placebo in 20 patients suffering from HS was included in our analysis (51). 10 patients received bermekimab and 10 were treated with placebo. The primary endpoint consisted of a positive hidradenitis suppurativa clinical response score (HiSCR). HiSCR was reached in 60% of the patients treated with bermekimab compared to 10% with placebo. This difference was significant. HiSCR at week 24 was 6 in the bermekimab arm compared to 0 in the placebo arm. The time to HS exacerbation was 11 weeks in the bermekimab group compared to seven weeks in the placebo group. There was a decrease in at least two of the assessed scores in 80% of patients in the bermekimab group compared to 40% in the placebo group. The decrease of total lesion depth was 77.8% in bermekimab versus 22% in placebo. These secondary endpoints were all non-significant.

There were less AEs observed in the bermekimab group (19 AEs) compared to the placebo group (24 AEs). This increase in AEs in the placebo arm was due to a higher number of HS exacerbations. The reported number of SAEs were similar in both study arms. QoL was significantly improved with bermekimab compared to placebo.

*Synopsis:* Both anakinra and bermekimab were safe and efficacious in treating patients suffering from HS. Larger RCTs are warranted to confirm these promising findings.

#### Hyper-IgD Syndrome

Hyper-IgD syndrome (HIDS) is caused by mevalonate kinase deficiency (MKD). MKD represents a spectrum of diseases, the clinical presentation of which depends on the amount of mevalonate kinase enzyme activity. Residual enzyme activity results in HIDS, whereas absent activity is seen in patients with mevalonate aciduria.

##### Anakinra

We found one observational, prospective case series that fulfilled our inclusion criteria (52). The efficacy of anakinra was investigated in patients with HIDS or MKD who received anakinra either continuously (one patient) or on demand during flares (10 patients), compared to an untreated control group. In total 20 patients were observed of which 11 received anakinra and nine received no treatment. Anakinra resulted in 72% clinical response – including a significant reduction of duration of fever, duration of symptoms, maximum temperature, and maximum CRP – compared to 0% in the control group. Due to the short duration of on-demand intervention, there were no AEs or SAEs reported. QoL was not investigated.

##### Canakinumab

De Benedetti et al. treated in the CLUSTER trial 72 patients suffering from HIDS (or MKD) with canakinumab (37 patients) or placebo (35 patients) and investigated the proportion of patients experiencing a complete response (37). 13 patients (35%) receiving canakinumab and only two patients (5.7%) receiving placebo showed a resolution of baseline symptoms and laboratory markers, thus fulfilling the criteria for a complete response. Regarding secondary outcomes, 46% of patients in the canakinumab group had a PGA score less than 2.0 compared to 6% assigned to placebo. The acute phase reactants CRP and SAA were significantly lower in the canakinumab group compared to placebo. Furthermore, the proportion of patients who did not have a flare after eight weeks of treatment was 50% in canakinumab and 14.3% in placebo. All these secondary endpoints were significant.

The number of AEs was significantly higher in patients treated with canakinumab (251 per 100 patient-years) compared to placebo (46 per 100 patient-years). Conversely, SAEs occurred in 11 cases receiving canakinumab versus three cases with placebo; this difference was due to a higher rate of HIDS flares, categorized as SAEs, in patients treated with placebo. QoL was not investigated.

*Synopsis:* Based on efficacy of canakinumab in HIDS and safety data that were comparable with standard-of-care treatment, canakinumab was approved by the EMA and FDA for the treatment of HIDS and MKD. Likewise, anakinra showed promising results in treating HIDS and MKD and, thus, may represent a possible on-demand treatment for this indication.

#### Macrophage Activation Syndrome

##### Anakinra

A phase-three RCT investigated the efficacy of anakinra in reducing mortality of macrophage activation syndrome (MAS), manifesting with hepato-biliary dysfunction and disseminated intravascular coagulopathy (53). During this 28-day follow-up study, 43 patients with MAS were randomized to receive either anakinra (26 patients) or placebo (17 patients) intravenously for 72 days. Mortality was significantly lower in the anakinra arm (34.6%) compared to placebo (64.7%), corresponding to a 47% reduction in mortality when treated with anakinra. The study did not investigate treatment-related AEs, SAEs or QoL.

*Synopsis:* A single RCT indicated a significant benefit of anakinra in reducing mortality of life-threatening MAS, associated with hepato-biliary dysfunction and disseminated intravascular coagulopathy.

#### Psoriasis Arthritis

##### Anakinra

One prospective, open-label, single-center study analyzing efficacy and safety of anakinra in psoriasis arthritis was included in our systematic review (54). 20 patients were included, of which only six (30%) completed the study period of six months, whereas 14 dropped out due to lack of efficacy. The 30% of patients completing the study showed a treatment response fulfilling the psoriasis arthritis response criteria (PsARC) in week 24, 25% in week four and 5% in week 12. Anakinra resulted in a reduction of the DAS28 of 20% in these six patients completing the study. Patients receiving anakinra experienced a total of 48 AEs of which three were SAEs. QoL was not investigated.

*Synopsis:* Based on this small open-label study in psoriasis arthritis, anakinra showed a favorable safety profile but no significant benefit.

#### Pyoderma Gangrenosum

##### Anakinra

A case series of three patients with refractory pyoderma gangrenosum (PG) investigated the effect of anakinra on duration to healing (55). The authors reported an average duration of 10.6 months (range 7-14 months) for complete healing of refractory PG lesions. AEs, SAEs, and QoL were not investigated.

##### Canakinumab

A case series of five patients with PG assessed treatment with canakinumab (56). After initiating therapy with canakinumab four out of five patients (80%) showed a complete clinical response, a sustained remission, and a reduction of PGA of at least one from baseline. The dermatology life quality index (DLQI) was reduced in four patients. Three patients showed a complete healing. The mean diameter of lesions was reduced from 4.3 cm at baseline to 0.7 cm at the last visit. Three AEs were reported of which one was categorized as a SAE.

*Synopsis:* Two small uncontrolled case series showed promising results with the use of anakinra and canakinumab in the treatment of PG. These findings need to be confirmed in RCTs.

#### Recurrent Pericarditis

##### Anakinra

One double-blind RCT investigated the number of recurrences of pericarditis and time to flare in 21 patients with recurrent pericarditis treated with anakinra or placebo during 12 months (57). In patients receiving anakinra recurrence of pericarditis only occurred in 18%, compared with 90% recurrence in the placebo group. The median time to a flare was 72 days in the placebo group and could not be calculated in the anakinra group since more than half of patients assigned to anakinra were still flare-free at the end of the study. Furthermore, all patients had a complete response to open-label anakinra treatment on day eight and all patients could withdraw GCs within six weeks of anakinra initiation, before entering the double-blind phase. There were 20 AEs and no SAEs reported. QoL and PGA were significantly improved with anakinra treatment.

##### Rilonacept

One RCT, fulfilling our inclusion criteria, investigated treatment of recurrent pericarditis with rilonacept (58). 86 patients with recurrent pericarditis were randomized but only 61 patients completed the run-in period. These patients received either rilonacept (30 patients) or placebo (31 patients) for 50 weeks in the withdrawal period. The primary endpoint was median time to pericarditis recurrence following withdrawal. Median time to pericarditis recurrence was significantly longer in the rilonacept group compared to placebo, with placebo resulting in 8.6 weeks, whereas the medium time to recurrence was incalculably long in the rilonacept group due to the very few pericarditis recurrences. 17 patients (56%) in the rilonacept group and four patients (13%) in the placebo arm remained in clinical remission at week 16 after treatment withdrawal. 97% of patients in the rilonacept arm stated they were pain-free for the study period compared to 45% in the placebo group. Moreover, 81% of patients receiving rilonacept had no symptoms compared to 25% with placebo. Furthermore, during the treatment run-in period time to pain response was five days, median time to normalization of CRP took seven days, and the time until GCs could fully be discontinued was 7.9 weeks in mean for the rilonacept group.

AEs occurred in 24 patients (80%) in the rilonacept group compared to 13 patients (42%) in the placebo group. This difference in AEs was significant. The amount of SAEs was similar in both study groups. QoL was not investigated.

*Synopsis:* Anakinra and rilonacept showed positive safety and efficacy data in the treatment of recurrent pericarditis. Thus, anakinra is a valuable therapeutic option in recurrent pericarditis, and rilonacept represents a possible treatment option in these patients.

#### Rheumatoid Arthritis

##### Anakinra

13 double-blind RCTs fulfilled our inclusion criteria concerning anakinra as a treatment for rheumatoid arthritis (RA) (59–71). Three trials, including one extension trial, investigated the use of anakinra in comparison to placebo (59, 61, 67). Nine trials, including one extension trial, investigated the use of anakinra in comparison to placebo plus DMARDs, non-steroidal anti-inflammatory drugs (NSAIDs) or GCs (33, 60, 62–65, 68). The most frequent DMARD was methotrexate at a stable dose between 7.5 and 25 mg weekly. Genovese et al. investigated efficacy of anakinra in combination with anti-tumor necrosis factor (anti-TNF) therapy with etanercept. The major inclusion criteria was active RA, diagnosed according to the ACR criteria. The most common exclusion criteria was an underlying autoimmune disease other than RA or systemic involvement of RA.

In most of the studies a combination of anakinra with either methotrexate or other DMARDs was superior to placebo plus methotrexate in terms of efficacy at 24 weeks (62, 64, 69, 70). Moreover, Bresnihan et al. (59) and Cohen et al. (60) showed more patients fulfilled the ACR20 response when increasing the dose of anakinra from 30 mg to 150 mg daily. Furthermore, Bresnihan et al. showed a significant reduction of 41% in the rate of radiological progression under treatment with anakinra compared to placebo (59). Nuki et al. showed in their extension phase, maintained improvement of 20% in the ACR response (ACR20) as well as of number of swollen tender joints, PGA, investigator’s global assessment (IGA), pain and health assessment questionnaires, and acute phase reactants for up to 48 weeks after ending the double-blind placebo-controlled phase, thus lending support for a long-term benefit of anakinra in RA (61).

Cohen et al. investigated the efficacy of anakinra plus methotrexate compared to placebo plus methotrexate by using the HAQ, which measures individual functional status (62). The study showed a rapid and considerable improvement in a dose-dependent manner. The two highest doses of anakinra (1 and 2mg/kg) even showed a statistically significant improvement of the HAQ compared to placebo. Genovese et al. studied the efficacy of anakinra in comparison to the anti-TNF agent etanercept (66). They demonstrated in all treatment arms an improvement of the ACR20, ACR50 and ACR70 responses from baseline at week 24, however, the combination of anakinra plus etanercept was not superior to etanercept alone. In fact, patients receiving etanercept only achieved the best ACR response of all groups.

The CARDERA-2 Trial evaluated differences in efficacy of anakinra plus methotrexate compared to methotrexate monotherapy focusing on radiologic changes and efficacy measure changes from baseline, including DAS28, HAQ, QoL, and ACR response rates (71). Where radiologic scores were similar in both treatment arms, methotrexate alone was superior to anakinra plus methotrexate in terms of DAS28, HAQ, QoL as well as ACR70. The opposite was seen in ACR20 and ACR50.

Three studies focused on safety of anakinra in RA investigating AEs and SAEs (63, 65, 68). Fleischmann et al. reported in total 1288 AEs in 92% of patients during the follow-up period of 24 weeks. Injection site reactions were the most common AEs. 87 SAEs (7.7%) in the anakinra group and nine SAEs (7.8%) in the placebo group were reported. The incidence of infectious episodes was similar in anakinra and placebo (63, 65, 68). However, serious infections, including most often pneumonia and cellulitis, were more frequent in the anakinra group (2.1%) than in the placebo group (0.4%) (65, 68).

##### Canakinumab

One RCT investigated the efficacy of canakinumab in patients with RA (72). Of 274 included patients, 183 finished the 12-week study and received canakinumab compared to 63 in the placebo group. The primary endpoint ACR50 after 12 weeks was reached by 26.5% in the arm receiving 150 mg canakinumab every four weeks compared to 11.4% in the placebo groups. Secondary endpoints compared the efficacy of different canakinumab dose regimens to placebo. The percentages of ACR70 responders in the 150 mg and 300 mg every four weeks were significantly higher than in placebo. The arm receiving 150 mg canakinumab every four weeks showed significant superiority in the DAS28, DAS-based EULAR criteria, PGA, and HAQ compared to placebo and other canakinumab dose regimens, such as 300 mg canakinumab every two weeks or 600 mg canakinumab intravenous loading plus 300 mg every two weeks. During the entire follow-up period, all three canakinumab treatment arms achieved a greater decrease in acute phase reactants compared to baseline.

52% of all patients treated with canakinumab experienced AEs compared to 53% in the placebo group. With 46.4% AEs, the group receiving 150 mg canakinumab every four weeks showed the lowest rate of all groups. SAEs occurred in 4.7% of canakinumab-treated individuals compared to 7.1% in the placebo group. Again, with 1.4%, the rate of SAEs was lowest in the group treated with 150 mg canakinumab every four weeks compared to all other groups.

*Synopsis:* Based on beneficial safety and efficacy data, use of anakinra was approved by the EMA and FDA for treating patients with RA. However, its use is limited to patients who fail to respond adequately to methotrexate alone (EMA) or to one or more DMARDs (FDA). Canakinumab demonstrated significant superiority over placebo in treating RA, which positions canakinumab as a viable alternative for the treatment of RA. Nevertheless, because of numerous other options for RA, no approval was sought by the EMA and FDA for this indication.

#### Schnitzler’s Syndrome

##### Anakinra

A total three case series assessed use of anakinra in a total of 27 patients with Schnitzler’s syndrome (73–75). Rowczenio et al. showed a treatment response and significant improvement of the mean QoL score in 19 out of 21 patients (90%) (73). Similar results were reported by Gran et al. in three patients and de Koning et al. in another three patients, demonstrating treatment responses of 100% (74, 75).

##### Canakinumab

We found one RCT fulfilling our inclusion criteria that analyzed the efficacy of treatment with canakinumab in Schnitzler’s syndrome (76). 20 patients suffering from Schnitzler’s syndrome were treated with either canakinumab or placebo in a 16-week trial. 71% achieved a complete clinical response after seven days with canakinumab compared to 0% with placebo. PGA was reduced by 11 points in canakinumab compared to 0 in placebo. CRP and SAA decreased by eight and 389, respectively, under treatment with canakinumab compared to zero and 13 with placebo. QoL improved in the canakinumab group (DLQI -5) and worsened in the placebo group (DLQI +0.5). There were no SAEs or AEs reported.

##### Rilonacept

One prospective, single-center, open-label study evaluated the use of rilonacept in eight patients with Schnitzler’s syndrome (77). The investigators showed that treatment with rilonacept resulted in a significant reduction of clinical symptoms compared to baseline, as measured with the Schnitzler’s activity score, a reduction of key symptoms, and an improvement of the PGA with a decrease from 6.5 to 3.5 points within 28 days.

The safety of rilonacept was also evaluated. 13 AEs occurred during treatment with rilonacept. There were no SAEs reported. The impact of rilonacept on QoL was not assessed in this study.

*Synopsis:* Evidence on their use in Schnitzler’s syndrome demonstrated very significant efficacy for anakinra and canakinumab in small case series and a small RCT, respectively. Rilonacept also showed improvement of clinical symptoms in a small uncontrolled and open-label study in patients with Schnitzler’s syndrome.

#### Sjögren’s Syndrome

##### Anakinra

One placebo-controlled RCT investigated Sjögren’s syndrome-associated fatigue in a total of 26 patients (78). There was no significant change in fatigue scores in both treatment groups at four weeks of treatment, although the investigators observed a reduction of fatigue scores of 30% in the anakinra and of 10% in the placebo group. Furthermore, fatigue levels were reported to return to baseline values one week after the last injection in both groups. Two AEs and no SAEs were reported, and QoL was not investigated.

*Synopsis:* Anakinra did not show a benefit in Sjögren’s syndrome-associated fatigue.

#### Synovitis, Acne, Pustulosis, Hyperhidrosis, Osteitis

##### Anakinra

We found only one case series meeting our criteria that described six patients suffering from synovitis, acne, pustulosis, hyperhidrosis, osteitis (SAPHO) treated with anakinra (79). This study showed a clinical response in five out of six (83%) patients, including improved disease activity as measured by a reduction of the visual acuity score (VAS) from 7.0 to 3.25 after one month and to 2.5 after five months. Furthermore, a reduction in axial involvement, inflammatory biomarkers and need of symptomatic treatments was demonstrated. Two AEs were reported, whereas no SAEs occurred.

*Synopsis:* The results reported with treating SAPHO with anakinra in the small group of patients were promising, thus warranting larger and controlled trials to confirm these findings.

#### Systemic Juvenile Idiopathic Arthritis

##### Anakinra

We found two RCTs investigating anakinra in systemic juvenile idiopathic arthritis (sJIA) (80, 81). 50 children of whom 11 suffered from sJIA were treated by Ilowite et al. with anakinra; due to lack of enrollment to meet the sample size required for statistical power, the study reported on safety instead of efficacy as the primary endpoint (80). Secondary endpoints included efficacy measures. Compared to placebo, anakinra showed a treatment response in 73% of patients resulted in a significant reduction in disease flares, with 22% of patients experiencing disease flares when treated with anakinra compared to 50% in the placebo group, both during the open-label phase of the study. Furthermore, patients receiving anakinra had a significantly longer time to flare and significant improvements of the HAQ and of laboratory markers of inflammation. Quartier et al. showed similar results with 67% of 24 patients responding to anakinra treatment compared to 8% with placebo (81). Compared to placebo, the anakinra group reached the modified ACR response more often, and disease activity was reduced by 63% in anakinra compared to 20% in the placebo group. AEs were not significantly different between anakinra and placebo, and injection site reactions represented the most frequent AEs (80, 81). Three SAEs were reported.

##### Canakinumab

We identified one RCT and its extension trial that fulfilled our inclusion criteria (82, 83). Thus, a first publication reported a phase-three study consisting of two consecutive trials (82). Trial number one (T1) included 84 patients observed for 29 days and trial number two (T2) assessed 100 patients until the first relapse. In both trials, patients were randomized at a 1:1 ratio to receiving either canakinumab or placebo. The primary endpoint of T1 was the JIA ACR30, which was reached by 36 patients (84%) treated with canakinumab compared to 4 patients (10%) in the placebo group. The primary endpoint of T2 was the median time to disease flare; the median time to disease flare could not be calculated in the canakinumab group because less than 50% of patients experienced a disease flare during the entire follow-up period of 617 days, whereas the median time to disease flare was 236 days in the placebo group. Secondary endpoints were significantly more frequently reached with canakinumab compared to placebo in both trials, including JIA ACR50 (canakinumab 82%, placebo 5%), JIA ACR70 (canakinumab 75%, placebo 2%), JIA ACR90 (canakinumab 61%, placebo 2%), JIA ACR100 (canakinumab 49%, placebo 2%), and inactive disease (canakinumab 46%, placebo 0%).

The extension trial of above-mentioned trials revealed that long-term efficacy was reached in patients treated with canakinumab in 73% of patients for JIA ACR50 after six months and in 54% for JIA ACR50 after three years (83). A complete response was reached in 18.6% of patients after six months and in 28% after three years. GC discontinuation was possible in 29% of patients after six months, 40% after two years, and 15.6% after five years. However, reoccurrence of disease activity was seen after withdrawal of canakinumab.

AEs were comparable in both study arms. Canakinumab was associated with 49 AEs in T1 and 272 AEs in T2 compared to placebo with 27 AEs in T1 and 229 AEs in T2. Likewise, similar frequencies of SAEs were noted in both study arms in T1 and T2.

##### Rilonacept

Two RCTs, fulfilling our inclusion criteria, assessed rilonacept in the treatment of patients with sJIA (84, 85). In a first RCT, 24 patients received either rilonacept (17 patients) or placebo (seven patients) (84). The primary endpoint was safety, whereas secondary endpoints consisted of clinical measures of disease and inflammation. During a four-week double-blind, placebo-controlled treatment period there were no significant differences measured in pediatric ACR30, ACR50 and ACR70 scores in patients receiving rilonacept compared to placebo. However, a greater proportion of patients in the rilonacept group experienced a reduction of clinical indicators of systemic inflammation. These reductions were maintained during the open-label 23-month phase. Thus, GC therapy could be discontinued, and PGA decreased from 5.5 to two points.

A second RCT (the RAPPORT trial) investigated rilonacept in 71 patients with sJIA (85). 35 patients received rilonacept versus 36 patients that were treated with placebo. 77% of patients in the rilonacept group showed a treatment response at week 12 compared to 59% with placebo, even though this was not statistically significant. However, pediatric ACR30, ACR50 and ACR70 scores were significantly lower in the rilonacept group compared to placebo. GCs were reduced more in the rilonacept arm than in placebo.

In terms of safety in the first RCT (84), only one AE (6%) was recorded in the rilonacept group compared to zero AEs in the placebo arm. During the double-blind period, there were no SAEs reported. In the open-label phase, three SAEs occurred. Also the second RCT found rilonacept to be safe, with 28% of patients in the rilonacept arm compared to 54% in the placebo arm experiencing AEs (85). Four SAEs in the rilonacept group and two in the placebo group were reported. QoL was not assessed in either of the two studies.

*Synopsis:* Based on positive safety and efficacy data, anakinra was approved by the EMA and canakinumab by both the EMA and FDA for the treatment of sJIA. Likewise, rilonacept was safe in patients with sJIA and one RCT demonstrated some efficacy in treating clinical symptoms of sJIA. Thus, rilonacept may represent a therapeutic option for sJIA.

#### TNF Receptor-Associated Periodic Syndrome

##### Anakinra

One case series assessing anakinra in five patients with confirmed TNF receptor-associated periodic syndrome (TRAPS) met our inclusion criteria (86). Following initiation of anakinra, all patients experienced a prompt disappearance of their symptoms and normalization of acute phase reactants, which was maintained during anakinra treatment during 4-20 months. However, after cessation of anakinra, all patients relapsed within three to eight days. No major AEs or SAEs were observed

##### Canakinumab

De Benedetti et al. treated in the CLUSTER trial 46 patients suffering from TRAPS with either canakinumab (22 patients) or placebo (24 patients) (37). The primary endpoint was the proportion of patients experiencing a complete response. 10 patients (45.4%) receiving canakinumab and zero patients (0%) treated with placebo showed a resolution of baseline symptoms and laboratory markers and consequently fulfilled the criteria for a complete response. Regarding secondary outcomes, 45% of patients in the canakinumab group had a PGA score less than two compared to 4% assigned to placebo. The acute phase reactants CRP and SAA were also significantly lower in the canakinumab group than in placebo. Moreover, the proportion of patients without a flare after eight weeks of treatment was 75% for canakinumab and 40% for placebo. All these secondary endpoints were significant.

The number of AEs was significantly higher in patients treated with canakinumab (112 per 100 patient-years) compared to placebo (46 per 100 patient-years). The rate of SAEs was similar in both study arms. In both groups three cases of SAEs occurred each. QoL was not investigated.

*Synopsis:* On the basis of convincing safety and efficacy data, canakinumab was approved by the EMA and FDA for the treatment of TRAPS. Anakinra also demonstrated a benefit in a small case series of TRAPS patients, thus it should be considered in patients with TRAPS.

#### Type 1 Diabetes Mellitus

##### Anakinra

There was one study meeting our inclusion criteria that assessed anakinra treatment in 69 patients with recent-onset type 1 diabetes mellitus (T1DM) (87). The primary endpoint of this phase-two clinical trial was endogenous production of stimulated C-peptide, which served as a surrogate marker for insulin production, after treatment with anakinra compared to placebo for nine months. Secondary endpoints included fasting glucose concentration, hemoglobin A1c (HbA1c), and the dose of insulin necessary over time. The investigators found no significant difference in stimulated C-peptide response between the two study groups. Furthermore, anakinra did not affect HbA1c, fasting glucose concentration, and the dose of insulin needed. 149 AEs were reported, which consisted mainly of injection site reactions, with 128 AEs occurring in the anakinra group and 21 in placebo, which indicated a significantly higher count of AEs with use of anakinra. No SAEs were reported.

##### Canakinumab

One RCT, fulfilling our inclusion criteria, assessed canakinumab as a treatment for recent-onset T1DM (87). This phase-two RCT evaluated treatment for nine months with either canakinumab (47 patients) or placebo (22 patients). The primary endpoint was the endogenous production of stimulated C-peptide, which served as a surrogate marker for intrinsic insulin production. Secondary endpoints were fasting glucose concentration, HbA1c, and necessary insulin dose over time. There was no significant difference found in stimulated C-peptide response between the two study groups. Furthermore, canakinumab did not affect the percentage of HbA1c, fasting glucose value, or the insulin dose needed. 121 cases of AEs were reported, with 81 AEs in the canakinumab group and 40 AEs in placebo, which results in similar frequencies of AEs in both treatment arms. There were two SAEs reported.

##### Gevokizumab

We identified one study, fulfilling our inclusion criteria that assessed gevokizumab as a treatment for recent-onset T1DM (88). This RCT included 26 patients, and its primary endpoint was endogenous production of stimulated C-peptide – as a surrogate marker for insulin production – after treatment with either gevokizumab or placebo for four months. Secondary endpoints were fasting glucose concentration, HbA1c, and necessary insulin dose over time. There was no significant difference found in stimulated C-peptide response between the two study groups. Moreover, gevokizumab did not affect the percentage of HbA1c, fasting glucose value, necessary insulin dose, or other laboratory values. 21 cases of AEs were reported, with five in placebo and 16 in the gevokizumab group, the latter of which was significantly higher than in placebo. There was one SAE reported in the gevokizumab group.

##### Rilonacept

One phase-one, prospective study assessing rilonacept in 13 T1DM patients fulfilled our inclusion criteria (89). The primary endpoint of this study was safety of rilonacept. The investigators recorded a total of 85 AEs. No SAEs were reported. Secondary outcomes, such as an effect of rilonacept on HbA1c or the necessary insulin dose over time could not be shown. QoL was not assessed.

*Synopsis:* IL-1-targeting biologics – including anakinra, canakinumab, gevokizumab, and rilonacept – showed no positive treatment effects on preserving pancreatic islet cell function and endogenous insulin production in patients with T1DM.

#### Urticarial Vasculitis

##### Canakinumab

A single-center, open-label, pilot trial tested canakinumab treatment in 10 patients suffering from urticarial vasculitis (90). Following initiation of canakinumab therapy, the urticarial vasculitis activity score (UVAS) was significantly reduced by a mean of 60% (-1.7 points) compared to baseline. Disease activity also decreased by 41%, with 40% of the patients demonstrating more than 50% improvement compared to baseline. A complete response was reached in 20%, and a mean improvement of 40% in QoL was observed in all patients. Nine AEs but no SAEs were recorded.

*Synopsis:* These findings suggested a beneficial role of canakinumab in treating urticarial vasculitis. Larger RCTs are needed to confirm these findings.

### Risk of Bias

We assessed the quality and risk of bias of the included studies using a modified Downs and Black checklist. The results of this analysis are available in **Table S5**.

## Discussion

The first IL-1-targeting biologic anakinra came in 2001 on the U.S. and in 2002 on the EU market, offering a novel and valuable treatment option for patients suffering from various autoinflammatory and autoimmune diseases. Since then, several additional IL-1-targeted biologics have been developed and assessed in different immune-mediated disorders. Consequently, the data on safety and efficacy of IL-1-targeting biologics in immune-mediated disorders have increased, thus making an overview on these outcome measures essential.

We summarized the current state of research and clinical efficacy of anakinra, bermekimab, canakinumab, gevokizumab, and rilonacept, an overview of which is provided in **Table 2**. As for the current evidence on safety of these IL-1-targeted biologics, we created a table summarizing the most frequent AEs reported in the analyzed studies fulfilling the criteria of inclusion for this systematic review (**Table 3**).

**Table 2:** Summary of the evidence.

		disease Still's Adult-onset	disease Behcet's	CAPS / MWS	DIRA	FMF	Gout	GvHD	suppurativa Hidradenitis	HIDS	MAS	arthritis Psoriasis	gangrenosum Pyoderma	pericarditis Recurrent	arthritis Rheumatoid	SAPHO	syndrome Schnitzler's	syndrome Sjögren's	sJIA	TRAPS	T1DM	vasculitis Urticarial
Biologics	Diseases																					
Anakinra		IV	IV	I	–	I	IIa	IIb	IIb	IIIb	IIb	IV	IV	IIb	I	IV	IV	IIb	I	IV	IIb	–
Bermekimab		–	–	–	–	–	–	–	IIIb	–	–	–	–	–	–	–	–	–	–	–	–	–
Canakinumab		IIb	IV	I	–	I	I	–	–	I	–	–	IV	–	IIb	–	IIb	–	I	I	IIb	IV
Gevokizumab		–	IIb	–	–	–	–	–	–	–	–	–	–	–	–	–	–	–	–	–	IIb	–
Rilonacept		IV	–	I	IV	IIb	IIb	–	–	–	–	–	–	IIb	–	–	IV	–	IIb	–	IIb	–
**Legend:**																						
Level I		Approved by the EMA and/or FDA																				
Level IIa		Multicentric double-blind RCT proving a significant superiority over standard-of-care treatment																				
Level IIb		Multicentric double-blind RCT proving a significant superiority over placebo																				
Level IIIa		Clinical study, not fulfilling the above-mentioned criteria, but proving a superiority over standard-of-care treatment																				
Level IIIb		Clinical study, not fulfilling the above-mentioned criteria, but proving a superiority over placebo																				
Level IV		Case series or open-label trials without control group with positive results																				
–		No or too little information available																				
Achieved	Failed		Mixed results																			

CAPS, cryopyrin-associated periodic syndromes; DIRA, deficiency in IL-1 receptor antagonist; EMA, European Medicines Agency; FDA, U.S. Food and Drug Administration; FMF, familial Mediterranean fever; GvHD, graft-versus-host disease; HIDS, hyper-IgD syndrome; MAS, macrophage activation syndrome; MWS, Muckle-Wells syndrome; SAPHO, synovitis, acne, pustulosis, hyperhidrosis, osteitis; sJIA, systemic juvenile idiopathic arthritis; TRAPS, TNF receptor-associated periodic syndrome; T1DM, type 1 diabetes mellitus.

Table 3Adverse events.

**Table:** 

Anakinra			
Organ systems affected	Adverse event(s)		Refs.
**Systemic**	**a) Immediate-type adverse reactions**	Fever	(25, 80, 81)
	**b) Infection**	Abscess, cellulitis, diverticulitis, ear infection, fungal skin infection, gangrene, gastrointestinal infection, herpes zoster, osteomyelitis, pneumonia, pyelonephritis, upper respiratory tract infection, urinary tract infection, yeast infection	(21, 25, 28–30, 35, 39, 52, 54, 57, 59, 61, 63–68, 73, 81, 87)
	**c) Neoplasm**	Adenocarcinoma of cecum, basal cell carcinoma, lymphoma, malignant melanoma, prostate carcinoma, pulmonary cancer, thyroid cancer, uterine carcinoma	(59, 61, 63, 64, 66)
**Cardiovascular**	Chest pain, ischemia, pulmonary artery hypertension		(21, 28, 57, 60, 66, 71, 87)
**Gastrointestinal and hepatic**	Abdominal discomfort, constipation, diarrhea, elevated liver enzymes, nausea and vomiting, oral ulcers, weight gain		(18, 25, 26, 28–30, 35, 39, 52, 54, 66, 68, 70, 78–81, 87)
**Hematologic events**	Anemia, eosinophilia, leukopenia, macrophage activation syndrome, thrombocytopenia		(28, 60, 61, 67, 70, 71, 87)
**Musculoskeletal**	Arthralgia, bone fracture, musculoskeletal pain		(18, 25, 35, 39, 54, 60, 66, 68, 71, 80, 81, 87)
**Nervous system (including eyes)**	Dizziness, fatigue, gait disturbance, headache, hypoesthesia, ocular hyperemia, sleep disorder, vertigo		(25, 28, 35, 39, 66, 68, 80, 81, 87)
**Renal and urogenital**	Hematuria, hyponatremia		(81, 87)
**Upper and lower airways**	Dyspnea, epistaxis, flue-like symptoms, pharyngitis, pneumonitis, rhinitis, sinusitis		(18, 25, 60, 65, 66, 68, 71, 80, 87)
**Skin**	Angioedema, injection site reaction, pruritus, oral aphtosis, rash, rosacea		(21, 25, 26, 28–30, 35, 39, 52, 54, 57, 59–61, 63–68, 71, 78–81, 86, 87)

**Table:** Bermekimab

Organ systems affected	Adverse event(s)		Refs.
**Systemic**	**a) Immediate-type adverse reactions**		
	**b) Infection** Cellulitis, influenza virus infection, upper respiratory infection, urinary tract infection		(91)
	**c) Neoplasm**		
**Cardiovascular**	Hypertension		(91)
**Gastrointestinal and hepatic**	Abdominal pain, constipation, diarrhea, nausea, toothache		(91)
**Hematologic events**			
**Musculoskeletal**	Muscle spasm, musculoskeletal pain		(91)
**Nervous system (including eyes)**	Fatigue, headache, ocular hyperemia, syncope		(91)
**Renal and urogenital**	Balanoposthitis, dysmenorrhea		(91)
**Upper and lower airways**			
**Skin**	Dry skin, erythema, hidradenitis, injection site reaction, pruritus, rash		(91)

**Table:** Canakinumab

Organ systems affected	Adverse event(s)		Refs.
**Systemic**	**a) Immediate-type adverse reactions**	Fever	(36, 37, 82, 83, 87)
	**b) Infection**	Bronchitis, cellulitis, cutaneous abscess, gastrointestinal infection, herpes infection, influenza virus infection, peritonitis, pneumonia, septic shock, upper respiratory tract infection, urinary tract infection, viral infection	(31, 32, 36, 37, 41–43, 72, 76, 82, 83, 87)
	**c) Neoplasm**		
**Cardiovascular**	Deep vein thrombosis, hypotension, hypertension, pericarditis, pulmonary hypertension		(19, 31, 37, 42, 43, 76, 82, 83, 87)
**Gastrointestinal and hepatic**	Abdominal pain, diarrhea, elevated liver enzymes, gastroenteritis, hepatitis, hepatobiliary disorder, nausea, weight gain		(19, 31, 36, 37, 42, 43, 72, 76, 82, 83, 87)
**Hematologic events**	Anemia, neutropenia, macrophage activation syndrome, thrombocytopenia		(36, 37, 83, 87)
**Musculoskeletal**	Arthralgia, joint pain, muscle spasm, musculoskeletal pain		(19, 31, 37, 42, 43, 76, 82, 87)
**Nervous system (including eyes)**	Fatigue, headache, vertigo, increased intraocular pressure, seizure, paraesthesia		(31, 32, 37, 42, 56, 82, 83, 87, 89)
**Renal and urogenital**	Hypokalemia		(37, 83)
**Upper and lower airways**	Asthma, cough, nasopharyngitis, rhinitis, sinus congestion		(31, 42, 43, 76, 82, 87)
**Skin**	Injection site reaction, pyoderma gangrenosum, rash, rosacea		(32, 36, 37, 42, 43, 56, 72, 76, 83, 87)

**Table:** Gevokizumab

Organ systems affected	Adverse event(s)		Refs.
**Systemic**	**a) Immediate-type adverse reactions**		
	**b) Infection**	Conjunctivitis, upper respiratory tract infection	(24, 88)
	**c) Neoplasm**		
**Cardiovascular**	Hypertension		(24)
**Gastrointestinal and hepatic**	Abdominal pain, constipation, diarrhea, dyspepsia, nausea		(24)
**Hematologic events**	Eosinophilia, neutropenia		(24)
**Musculoskeletal**	Arthralgia, back pain, musculoskeletal pain		(24)
**Nervous system (including eyes)**	Blurred vision, cataract, depression, dizziness, eye pain, fatigue, headache, hypoaesthesia, macular edema		(24, 88)
**Renal and urogenital**			
**Upper andlower airways**	Cough		(24)
**Skin**	Face swelling		(24)

**Table:** Rilonacept

Organ systems affected	Adverse event(s)		Refs.
**Systemic**	**a) Immediate-type adverse reactions**	Allergic reaction, fever	(34, 58, 84, 85)
	**b) Infection**	Liver abscess, otitis media, pneumonia, skin infection, upper respiratory tract infection, urinary tract infection, vaginal infection, viral gastroenteritis	(33, 34, 38, 44–47, 58, 77, 84, 85, 89)
	**c) Neoplasm**	Actinic keratosis, basal cell carcinoma, prostate cancer, squamous cell carcinoma	(45, 58, 77)
**Cardiovascular**	Carotid artery dissection, palpitation, pericarditis		(58, 85)
**Gastrointestinal and hepatic**	Abdominal pain, diarrhea, dyspepsia, ileus, liver function abnormalities, nausea		(33, 45, 47, 58, 85, 89)
**Hematologic events**	Eosinophilia, neutropenia, macrophage activation syndrome, pancytopenia		(84)
**Musculoskeletal**	Arthralgia, gout exacerbation, joint pain, musculoskeletal pain		(33, 44, 45, 77, 84, 85, 89)
**Nervous system (including eyes)**	Depression, dizziness, fatigue, headache, hordeolum, hypoesthesia, stroke		(33, 44, 46–48, 58, 85)
**Renal and urogenital**	Altered renal function		(45)
**Upper and lower airways**	Cough, pulmonary fibrosis, rhinorrhea		(33, 34, 44, 84, 85, 89)
**Skin**	Angioedema, eczema, erythema, hair loss, hyperhidrosis, injection site reaction, plantar wart, rash		(33, 34, 38, 44–47, 58, 77, 84, 89)

Based on favorable safety and efficacy data, anakinra has been approved by the EMA and FDA for treating patients with CAPS, FMF, RA, and sJIA. Furthermore, anakinra demonstrated promising safety and efficacy results in RCTs in gout, MAS, and recurrent pericarditis. Contrarily, treatment with anakinra did not significantly improve the investigated parameters in AOSD, GvHD, SjS, and T1DM.

Convincing safety and efficacy data have resulted in the approval of canakinumab by the EMA and FDA for treating patients with CAPS/MWS, FMF, gout, HIDS, sJIA, and TRAPS. Moreover, canakinumab revealed promising safety and efficacy data in RCTs in RA and Schnitzler’s syndrome. Conversely, results with canakinumab in the treatment of AOSD and T1DM were rather negative.

On the basis of positive safety and efficacy results, rilonacept has been approved by the EMA and FDA for treating patients with CAPS/MWS. Furthermore, rilonacept demonstrated promising safety and efficacy results in RCTs in FMF, recurrent pericarditis, and sJIA. However, rilonacept failed to significantly improve surrogate markers of T1DM.

One RCT meeting our inclusion criteria showed mixed results in treating BD-associated uveitis with gevokizumab. These findings might warrant larger RCTs assessing gevokizumab or other IL-1-targeted biologics in BD-associated uveitis. However, whether IL-1-targeting biologics could benefit systemic BD cannot be concluded from this trial in BD-associated uveitis, as the ‘immune privileged’ microenvironment of the inner eye is different from the rest of the body (92). Gevokizumab is currently not approved by the EMA or FDA for any indication, nor is it available on the market.

A small RCT demonstrated the safety and efficacy of bermekimab in treating HS. Larger RCTs are warranted to confirm these findings. Bermekimab is currently not approved by the EMA or FDA and not on the market.

## Limitations

We applied standardized systematic review techniques in order to minimize the risk of bias. Additionally, the modified Downs and Black checklist was used to assess the quality and risk of bias of each study analyzed (**Table S2**).

Nevertheless, our systematic review has several limitations. The included studies had different outcome measures, premedications, inclusion criteria, concomitant treatments, durations, and control groups, which rendered a direct comparison difficult. Furthermore, small case series and open label trials were also included in our analysis, thus the reported results may be influenced by chance and the findings may not be as reliable as when obtained in large, double-blind RCTs.

## Conclusion

This study of IL-1-targeting biologics summarizes the current state of research, safety, and clinical efficacy of anakinra, bermekimab, canakinumab, gevokizumab, and rilonacept in treating immune-mediated disorders. Our systematic review showed that IL-1-targeted biologics are safe and effective in treating CAPS/MWS, DIRA, FMF, gout, HIDS, HS, MAS, PG, RA, recurrent pericarditis, SAPHO, Schnitzler’s syndrome, sJIA, and TRAPS. Conversely, IL-1-targeting biologics demonstratred only partial efficacy or failed to show superiority over placebo or standard-of-care treatment in AOSD, BD, GvHD, psoriasis arthritis, SjS, T1DM, and urticarial vasculitis. These findings will serve as a resource for clinicians and researchers, advancing research and development of IL-1-targeted biologics in immune-mediated disorders.

## Data Availability Statement

The original contributions presented in the study are included in the article/**Supplementary Material**. Further inquiries can be directed to the corresponding author.

## Author Contributions

Conception and design of the work: DA and OB. Data collection: DA and AY. Data analysis and interpretation: DA. Drafting the article: DA and OB. Critical revision of the article and final approval of the version to be published: DA, AY, and OB. All authors contributed to the article and approved the submitted version.

## Funding

This work was funded by the Clinical Research Priority Program of the University of Zurich for CRPP CYTIMM-Z (to OB).

## Conflict of Interest

The authors declare that the research was conducted in the absence of any commercial or financial relationships that could be construed as a potential conflict of interest

## Publisher’s Note

All claims expressed in this article are solely those of the authors and do not necessarily represent those of their affiliated organizations, or those of the publisher, the editors and the reviewers. Any product that may be evaluated in this article, or claim that may be made by its manufacturer, is not guaranteed or endorsed by the publisher.

## Abbreviations

ACR, American College of Rheumatology; AE, adverse event; AOSD, adult-onset Still’s disease; BD, Behçet’s disease; CAPS, cryopyrin-associated periodic syndromes; CRP, C-reactive protein; DAS, disease activity score; DAS28, 28-joint disease activity score; DIRA, deficiency in IL-1 receptor antagonist; DLQI, dermatology life quality index; DMARD, disease-modifying antirheumatic drug; eGFR, estimated glomerular filtration rate; EMA, European Medicines Agency; ESR, erythrocyte sedimentation rate; EULAR, European League Against Rheumatism; FDA, U.S. Food and Drug Administration; Fc, fragment crystallizable; FMF, familial Mediterranean fever; GC, glucocorticoid; GvHD, graft-versus-host disease; HAQ, health assessment questionnaire; HbA1c, hemoglobin A1c; HIDS, hyper-IgD syndrome; HiSCR, hidradenitis suppurativa clinical response; HS, hidradenitis suppurativa; IGA, investigator’s global assessment; IL, interleukin; IL-1 receptor antagonist; mAb, monoclonal antibody; MAS, macrophage activation syndrome; MKD; mevalonate kinase deficiency; MWS, Muckle-Wells syndrome; NSAID non-steroidal anti-inflammatory drug; PAPA, pyogenic arthritis, pyoderma gangrenosum and acne; PFAPA, periodic fever, aphthous stomatitis, pharyngitis, and adenitis; PDN, prednisone; PG, pyoderma gangrenosum; PGA, patient global assessment; PsARC, psoriasis arthritis response criteria; QoL, quality of life; RA, rheumatoid arthritis; RCT, randomized controlled trial; SAA, serum amyloid A; SAE, serious adverse event; SAPHO, synovitis, acne, pustulosis, hyperhidrosis, osteitis; SC, subcutaneous(ly); SF-36, 36-Item Short Form Survey; sJIA, systemic juvenile idiopathic arthritis; SjS, Sjögren’s syndrome; SLE, systemic lupus erythematosus; T1DM, type 1 diabetes mellitus; TNF, tumor necrosis factor; TRAPS, TNF receptor-associated periodic syndrome; UVAS, urticarial vasculitis activity score; VAS, visual acuity score

## Glossary

**Table:**

ACR	American College of Rheumatology
AE	adverse event
AOSD	adult-onset Still’s disease
BD	Behçet's disease
CAPS	cryopyrin-associated periodic syndromes
CRP	C-reactive protein
DAS	disease activity score
DAS28	28-joint disease activity score
DIRA	deficiency in IL-1 receptor antagonist
DLQI	dermatology life quality index
DMARD	disease-modifying antirheumatic drug
eGFR	estimated glomerular filtration rate
EMA	European Medicines Agency
ESR	erythrocyte sedimentation rate
EULAR	European League Against Rheumatism
FDA	U.S. Food and Drug Administration
Fc	fragment crystallizable
FMF	familial Mediterranean fever
GC	glucocorticoid
GvHD	graft-versus-host disease
HAQ	health assessment questionnaire
HbA1c	hemoglobin A1c
HIDS	hyper-IgD syndrome
HiSCR	hidradenitis suppurativa clinical response
HS	hidradenitis suppurativa
IGA	investigator's global assessment
IL	interleukin
IL-1	receptor antagonist
mAb	monoclonal antibody
MAS	macrophage activation syndrome
MKD	mevalonate kinase deficiency
MWS	Muckle-Wells syndrome
NSAID	non-steroidal anti-inflammatory drug
PAPA	pyogenic arthritis, pyoderma gangrenosum and acne
PFAPA	periodic fever, aphthous stomatitis, pharyngitis, and adenitis
PDN	prednisone
PG	pyoderma gangrenosum
PGA	patient global assessment
PsARC	psoriasis arthritis response criteria
QoL	quality of life
RA	rheumatoid arthritis
RCT	randomized controlled trial
SAA	serum amyloid A
SAE	serious adverse event
SAPHO	synovitis, acne, pustulosis, hyperhidrosis, osteitis
SC	subcutaneous(ly)
SF-36	36-Item Short Form Survey
sJIA	systemic juvenile idiopathic arthritis
SjS	Sjögren’s syndrome
SLE	systemic lupus erythematosus
T1DM	type 1 diabetes mellitus
TNF	tumor necrosis factor
TRAPS	TNF receptor-associated periodic syndrome
UVAS	urticarial vasculitis activity score
VAS	visual acuity score
