# Supplementary material for: Systematic Review of Safety and Efficacy of IL-1-Targeted Biologics in Treating Immune-Mediated Disorders
Source: Front Immunol. 2022 Jul 6;13:888392. doi: 10.3389/fimmu.2022.888392 (PMC9296857; doi:10.3389/fimmu.2022.888392)
Supplement: Supplementary file 1 [file Table_1.pdf]

Table S1. Search terms.

1	Anakinra	6302
2	Anakinra AND adult's onset Still’s disease	141
3	Anakinra AND adult's onset Still’s disease, filters: RCT	2
4	Anakinra AND Behcet	62
5	Anakinra AND CAPS	150
6	Anakinra AND cryopyrin-associated periodic syndromes	136
7	Anakinra AND deficiency in IL-1 receptor antagonist	265
8	Anakinra AND DIRA	23
9	Anakinra AND type 1 diabetes	44
10	Anakinra AND type 1 diabetes, filters: RCT	1
11	Anakinra AND familial Mediterranean fever	146
12	Anakinra AND FMF	98
13	Anakinra AND familial Mediterranean fever, filters: RCT	2
14	Anakinra AND gout	89
15	Anakinra AND gout, filters: RCT	2
16	Anakinra AND graft versus host disease	13
17	Anakinra AND GVHD	7
18	Anakinra AND graft versus host disease, filters: RCT	1
19	Anakinra AND hidradenitis suppurativa	26
20	Anakinra AND acne inversa	26
21	Anakinra AND hidradenitis suppurativa, filters: RCT	1
22	Anakinra AND hyper IgD syndrome	57
23	Anakinra AND HIDS	27
24	Anakinra AND macrophage activation syndrome	93
25	Anakinra AND macrophage activation syndrome, filters: RCT	2
26	Anakinra AND Muckle Wells syndrome	177
27	Anakinra AND Periodic fever aphtous stomatitis pharyngitis adenitis syndrome	9
28	Anakinra AND PFAPA	12
29	Anakinra AND psoriasis arthritis	38
30	Anakinra AND psoriasis	98
31	Anakinra AND pyoderma gangrenosum	39
32	Anakinra AND pyogenic arthritis, pyoderma gangrenosum, acne	16
33	Anakinra AND PAPA	32
34	Anakinra AND recurrent idiopathic pericarditis	41
35	Anakinra AND pericarditis	71
36	Anakinra AND recurrent idiopathic pericarditis, filters: RCT	1
37	Anakinra AND relapsing polychondritis	8
38	Anakinra AND polychondritis	9
39	Anakinra AND rheumatoid arthritis	661
40	Anakinra AND rheumatoid arthritis, filters: RCT	36
41	Anakinra AND Schnitzler syndrome	114
42	Anakinra AND Sjögren syndrome	18
43	Anakinra AND Sjögren syndrome, filters: RCT	1
44	Anakinra AND synovitis, acne, pustulosis, hyperhidrosis, osteitis	0
45	Anakinra AND SAPHO	14
46	Anakinra AND systemic juvenile idiopathic arthritis	151
47	Anakinra AND systemic juvenile idiopathic arthritis, filters: RCT	2
48	Anakinra AND systemic sclerosis	18
49	Anakinra AND TNF receptor-associated periodic syndrome	30
50	Anakinra AND TRAPS	57
51	Anakinra AND urticarial vasculitis	7

1	Canakinumab	780
2	Canakinumab AND adult's onset Still’s disease	34
3	Canakinumab AND adult's onset Still’s disease, filters: RCT	1
4	Canakinumab AND Behcet	36
5	Canakinumab AND CAPS	110
6	Canakinumab AND cryopyrin-associated periodic syndromes	115
7	Canakinumab AND CAPS, filter: RCT	4
8	Canakinumab AND deficiency in IL-1 receptor antagonist	10
9	Canakinumab AND DIRA	7
10	Canakinumab AND type 1 diabetes	4
11	Canakinumab AND type 1 diabetes, filters: RCT	1
12	Canakinumab AND familial Mediterranean fever	89
13	Canakinumab AND FMF	67
14	Canakinumab AND familial Mediterranean fever, filters: RCT	3
15	Canakinumab AND gout	73
16	Canakinumab AND gout, filters: RCT	6
17	Canakinumab AND hidradenitis suppurativa	5
18	Canakinumab AND acne inversa	5
19	Canakinumab AND hyper IgD syndrome	28
20	Canakinumab AND HIDS	10
21	Canakinumab AND hyper IgD syndrome, filter: RCT	1
22	Canakinumab AND macrophage activation syndrome	32
23	Canakinumab AND macrophage activation syndrome, filters: RCT	3
24	Canakinumab AND Muckle Wells syndrome	103
25	Canakinumab AND Muckle Wells syndrome, filter: RCT	4
26	Canakinumab AND periodic fever aphtous stomatitis pharyngitis adenitis syndrome	4
27	Canakinumab AND PFAPA	4
28	Canakinumab AND psoriasis arthritis	9
29	Canakinumab AND psoriasis	13
30	Canakinumab AND pyoderma gangrenosum	12
31	Canakinumab AND pyogenic arthritis, pyoderma gangrenosum, acne	5
32	Canakinumab AND PAPA	8
33	Canakinumab AND recurrent idiopathic pericarditis	8
34	Canakinumab AND pericarditis	12
35	Canakinumab AND rheumatoid arthritis	58
36	Canakinumab AND rheumatoid arthritis, filters: RCT	3
37	Canakinumab AND Schnitzler syndrome	18
38	Canakinumab AND Schnitzler syndrome, filters: RCT	2
39	Canakinumab AND synovitis, acne, pustulosis, hyperhidrosis, osteitis	0
40	Canakinumab AND SAPHO	3
41	Canakinumab AND systemic juvenile idiopathic arthritis	77
42	Canakinumab AND systemic juvenile idiopathic arthritis, filters: RCT	6
43	Canakinumab AND TNF receptor-associated periodic syndrome	13
44	Canakinumab AND TRAPS	26
45	Canakinumab AND TRAPS, filter: RCT	1
46	Canakinumab AND urticarial vasculitis	2

1	Rilonacept	178
2	Rilonacept AND adult's onset Still’s disease	15
3	Rilonacept AND Behcet	4
4	Rilonacept AND CAPS	47
5	Rilonacept AND cryopyrin-associated periodic syndromes	41
6	Rilonacept AND CAPS, filter: RCT	1
7	Rilonacept AND deficiency in IL-1 receptor antagonist	8
8	Rilonacept AND DIRA	6
9	Rilonacept AND type 1 diabetes	3
10	Rilonacept AND type 1 diabetes, filters: RCT	1
11	Rilonacept AND familial Mediterranean fever	33
12	Rilonacept AND FMF	23
13	Rilonacept AND familial Mediterranean fever, filters: RCT	5
14	Rilonacept AND gout	42
15	Rilonacept AND gout, filters: RCT	6
16	Rilonacept AND hidradenitis suppurativa	2
17	Rilonacept AND acne inversa	2
18	Rilonacept AND hyper IgD syndrome	5
19	Rilonacept AND HIDS	5
20	Rilonacept AND macrophage activation syndrome	6
21	Rilonacept AND Muckle Wells syndrome	36
22	Rilonacept AND Muckle Wells syndrome, filter: RCT	1
23	Rilonacept AND periodic fever aphtous stomatitis pharyngitis adenitis syndrome	3
24	Rilonacept AND PFAPA	3
25	Rilonacept AND psoriasis arthritis	2
26	Rilonacept AND psoriasis	3
27	Rilonacept AND pyoderma gangrenosum	2
28	Rilonacept AND pyogenic arthritis, pyoderma gangrenosum, acne	1
29	Rilonacept AND PAPA	2
30	Rilonacept AND recurrent idiopathic pericarditis	10
31	Rilonacept AND pericarditis	27
32	Rilonacept AND pericarditis, filters: RCT	2
33	Rilonacept AND rheumatoid arthritis	20
34	Rilonacept AND Schnitzler syndrome	6
35	Rilonacept AND synovitis, acne, pustulosis, hyperhidrosis, osteitis	0
36	Rilonacept AND SAPHO	1
37	Rilonacept AND systemic juvenile idiopathic arthritis	28
38	Rilonacept AND systemic juvenile idiopathic arthritis, filters: RCT	6
39	Rilonacept AND TNF receptor-associated periodic syndrome	3
40	Rilonacept AND TRAPS	8
41	Rilonacept AND systemic sclerosis	3

1	Gevokizumab	51
2	Gevokizumab AND Behcet	15
3	Gevokizumab AND Behcet, filters: RCT	2
4	Gevokizumab AND type 1 diabetes	3
5	Gevokizumab AND type 1 diabetes, filters RCT	1
6	Gevokizumab AND psoriasis	2

1	Bermekimab OR MABp1	49
2	Bermekimab OR MABp1 AND hidradenitis suppurativa	5
3	Bermekimab OR MABp1 AND hidradenitis suppurativa, filters: RCT	3
