# Supplementary material for: Systematic Review of Safety and Efficacy of IL-1-Targeted Biologics in Treating Immune-Mediated Disorders
Source: Front Immunol. 2022 Jul 6;13:888392. doi: 10.3389/fimmu.2022.888392 (PMC9296857; doi:10.3389/fimmu.2022.888392)
Supplement: Supplementary file 3 [file Table_3.pdf]

Table S3. Specific reasons for exclusion.

Full-text articles excluded not matching inclusion criteria (n = 404)

ANK (n = 273)

Different disease or study drug (n = 97)
Non-RCT (n = 7)
Others (n = 20)
Post-hoc analysis (n = 1)
Retrospective analysis (n = 3)
Review (n = 5)
Study size less than 3 patients (n = 99)
Study without primary endpoint of interest (n = 41)

BER (n = 2)

Non RCT (n = 2)

CAN (n = 55)

Different disease or study drug (n = 21)
Non-RCT (n = 4)
Other (n = 3)
Retrospective analysis (n = 3)
Review (n = 3)
Study included two diseases of interest (n = 3)
Study size less than 3 patients (n = 11)
Study without primary endpoint of interest (n = 7)

GEV (n = 4)

Different disease or study drug (n = 2)
Non-RCT (n = 1)
Study size less than 3 patients (n = 1)

RIL (n = 70)

Different disease or study drug (n = 9)
Non-RCT (n = 3)
Others (n = 3)
Review (n = 48)
Study size less than 3 patients (n = 1)
Study without primary endpoint of interest (n = 6)

Abbreviations:
ANK, anakinra; BER, bermekimab; CAN, canakinumab; GEV, gevokizumab; RCT, randomized controlled trial; RIL, rilonacept,
