# Supplementary material for: Systematic Review of Safety and Efficacy of IL-1-Targeted Biologics in Treating Immune-Mediated Disorders
Source: Front Immunol. 2022 Jul 6;13:888392. doi: 10.3389/fimmu.2022.888392 (PMC9296857; doi:10.3389/fimmu.2022.888392)
Supplement: Supplementary file 4 [file Table_4.pdf]

Table S4. Main characteristics of included studies.

ANAKINRA (ANK)											
**Adult-onset Still's disease (AOSD)**											
Source	Patients treated	Verum	Primary endpoint (1°) investigated	Secondary endpoint (2°) investigated	Verum reaching 1°	Control reaching 1°	Verum reaching 2°	Control reaching 2°	Adverse events (AEs) verum	QoL	Serious adverse events (SAEs)
Nordström et al. (2012)	22	ANK 100 mg daily	remission according to specific criteria at 8 weeks	no secondary endpoint	7 patients (58%) reaching remission	2 patients (20%) reaching remission	no information	no information	3	no information	0
**Behcet's disease (BD)**											
Grayson et al. (2017)	6	ANK 100, 200 or 300 mg daily	complete response (absence of oral and genital ulcers on physical examination on two consecutive monthly visits between months 3 and 6)	number of physician-observed ulcers, number and severity of patient-reported ulcers, patient and PGA, and standardized DAS	33%	no control	no significant changes	no control	34	no significant changes	4
Cantarini et al. (2013)	9	ANK 100–150 mg daily	initial treatment response	occurrence of relapse	77.8%	no control	88%	no control	4	no information	0

Cryopyrin-associated periodic syndrome (CAPS)											
Source	Patients treated	Verum	Primary endpoint (1°) investigated	Secondary endpoint (2°) investigated	Verum reaching 1°	Control reaching 1°	Verum reaching 2°	Control reaching 2°	Adverse events (AEs) verum	QoL	Serious adverse events (SAEs)
Kullenberg et al. (2016)	43	ANK 1.5-2.5 mg/kg daily	number of AEs	no secondary endpoint	95%	no control	no information	no control	1233	no information	24 SAEs (cellulitis, wound infection, chest pain, uveitis, gastroenteritis, MAS, meningitis, arthritis, lymphadenitis, pneumonia sinusitis, convulsion)
Lepore et al. (2010)	20	ANK 1 mg/kg/d (max, 100 mg)	health-related QoL with 50-item version of the Child Health Questionnaire (CHQPF50)	no secondary endpoint	physical score: pre-treatment: median, 38. posttreatment: 52.2 (P < .001); psychological score: pre-treatment: 43.8, post-treatment 47.8; (P < .05)	no information	no information	no information	12	dramatic, significant and sustained amelioration of QoL, greater improvement physical score than in the psychosocial score	0
Eskola et al. (2018)	3	ANK 2 mg/kg/day, 3.5 mg/kg/day	clinical and laboratory response	no secondary endpoint	30%	no control	no secondary endpoint	no control	no information	no information	0
Sibley et al. (2013)	26	ANK 1mg/kg/d, 0.5-1mg/kg increase per	Proportion of patients with clinical and laboratory	organ specific outcomes (CNS, vision,	6m 100%, 12m 46% 24m 50%,	no control	No Indicators of active CNS compared to baseline.	no control	584	no information	6 (wound infections, MAS, hypopyon,

		injection (max 5 mg/kg/d)	response to ANK at 6, 12, 18, 24, 30, and 36 months	hearing, bone)	36m 58% and 60m 65%		Improvement in hearing occurred in 30% and progression of hearing loss was halted in the majority of the patients. Visual acuity and peripheral vision improved or stabilized in most patients over 5 years. Despite ANK therapy, the volume of the bony lesions increased significantly.				vertigo, gastroenteritis)
**Familial Mediterranean fever (FMF)**											
Source	Patients treated	Verum	Primary endpoint (1°) investigated	Secondary endpoint (2°) investigated	Verum reaching 1°	Control reaching 1°	Verum reaching 2°	Control reaching 2°	Adverse events (AEs) verum	QoL	Serious adverse events (SAEs)
Ben-Zvi et al. (2017)	25	ANK 100 mg daily	change in total number of attacks over the study period, adjusted to participation time (number of attacks per patient per month)	number of attacks per FMF site (abdominal, chest, joint, skin), levels of acute phase reactants, QoL	reduction of the number of attacks -66%	reduction of the number of attacks -20%	reduction of number of attacks per FMF site -30%, reduction of levels of acute phase reactants -90%, improvement of QOL +50%	no reduction of number of attacks per FMF site, reduction of levels of acute phase reactants -50%, worsening of QoL -10%	94	significant improvement in QoL with ANK by 50% compared to worsening by 10% in placebo	0 vs. 0

Gout											
Source	Patients treated	Verum	Primary endpoint (1°) investigated	Secondary endpoint (2°) investigated	Verum reaching 1°	Control reaching 1°	Verum reaching 2°	Control reaching 2°	Adverse events (AEs) verum	QoL	Serious adverse events (SAEs)
Janssen et al. (2018)	88	ANK 100 mg once daily for 5 days	mean change in patient-reported pain in the most affected joint from baseline at days 2 and 4	reduction of score in joint tenderness, joint swelling, CRP values, treatment response score	-75%	-60%	no difference in the natural course of symptom reduction under both therapeutic regimens		15	no information	0 vs. 0
**Graft-versus-host disease (GvHD)**											
Antin et al. (2002)	186	ANK 0.5 mg/kg per hour by continuous IV infusion from day -4 through 10	the development of grades B-D acute GVHD within 100 days of stem cell infusion	survival at day 100, incidence of transpl. related toxicities (eg, hepatic veno-occlusive disease, interstitial pneumonitis), and time to engraftment	61%	59%	survival day 100 74% , complications 29% (hepatic venoocclusive disease), time to engraftment 24 days	survival day 100 76% , complications 27% (hepatic venoocclusive disease), time to engraftment 23.5 days	no information	no information	no information
**Hidradenitis suppurativa (HS)**											
Tzatanekou et al. (2017)	20	ANK 100 mg	effect of ANK on HS disease severity: number of patients with reduction of DAS	time to a new exacerbation	78% of patients	20% of patients	Time to 50% of patients experiencing a relapse: more than 24 weeks of follow-up period	Time to 50% of patients experiencing a relapse: 6 weeks,	3	The change in overall DLQI at weeks 12 and 24 from baseline at week 0 was not different between the study arms	0 vs. 0
**Hyper-IgD syndrome (HIDS)**											

Source	Patients treated	Verum	Primary endpoint (1°) investigated	Secondary endpoint (2°) investigated	Verum reaching 1°	Control reaching 1°	Verum reaching 2°	Control reaching 2°	Adverse events (AEs) verum	QoL	Serious adverse events (SAEs)
Bodar et al. (2011)	11	ANK continuous (1–2 mg/kg/day) or on-demand treatment (100 mg/day or 1 mg/kg/day for 5–7 days).	clinical response	duration of fever (days), duration of symptoms (days), maximum temperature, maximum CRP	72%	0%	fever days 2.2, symptom days 3.8, max. temp. 38.4, max. CRP 80	fever days 6.3, symptom days 7.3, max. temp. 39.6, max. CRP 150	0	no information	0
**Macrophage activation syndrome (MAS)**											
Shakoory et al. (2016)	43	ANK 2.0 mg/kg/hr for 72 hours	28-day survival (mortality)	no information	mortality 34.6%	mortality 64.7%	no information	no information	did not reveal either increased mortality rate or AEs/SAEs over placebo	no information	0
**Muckle-Wells syndrome (MWS)**											
Kuemmerle et al. (2013)	26	ANK 1 to 2 mg/kg/day in patients <40 kg body weight and at a dose of 100 mg/day for those of ≥40 kg body weight	clinical disease activity as determined by MWS-DAS	percentage of patients achieving remission, inflammatory markers (ESR, CRP, SAA, S100A12)	MWS-DAS from 13 to 3 (P <0.001)	MWS-DAS from 6 to 3 (P = 0.002).	75% in remission, elevated ESR (8 to 8%), elevated CRP (92 to 25%), elevated SAA (92 to 25%), elevated S100A12 (55 to 45%),	93% in remission, elevated ESR (64 to 7%), elevated CRP (86 to 7%), elevated SAA (64 to 7%), elevated S100A12 (71 to 14%),	9	no information	0 vs. 1
Kuemmerle et al. (2011)	12	ANK 1–2 mg/kg in patients weighing 40 kg and 100 mg for patients	response to ANK therapy at 2 weeks of treatment (DAS for MWS of 10 at 2 weeks)	efficacy at the primary study end point (2 weeks) and long-term efficacy (MWS-DAS, markers of	100%	no control	overall disease activity decreased from 6.3 to 2.8 (P = 0.0005), Classic	no control	14	significant and sustained positive impact on the QoL	0

		weighing 40 kg		imflammation ,			markers of inflammation improved in 91%				
**Psoriasis arthritis**											
Source	Patients treated	Verum	Primary endpoint (1°) investigated	Secondary endpoint (2°) investigated	Verum reaching 1°	Control reaching 1°	Verum reaching 2°	Control reaching 2°	Adverse events (AEs) verum	QoL	Serious adverse events (SAEs)
Jung et al. (2010)	20	ANK 100 mg daily	AE profile and fulfilling Psoriasis Arthritis Response Criteria (PsARC)	DAS 28, ACR, EULAR, PASI Score, Dactylitis Score and (HAQ), and the CRP and ESR	31%	no control	4.7 to 4.0 DAS 1.12 to 1.05, HAQ, 20.4 to 26.4 CRP, 18.9 to 17.6 ESR at Week 0 to Week 24	no control	45	no information	3, a fracture of pubic bone, a increase in liver enzymes erysipel
**Pyoderma gangrenosum (PG)**											
Beynon et al. (2017)	3	ANK 100 mg	time to healing of refragtory PG after initiation of ANK	no secondary endpoint	(1) 14 months, (2) 7 months, (3) 11 months	no control	no secondary endpoint	no control	no information	no information	0
**Recurrent pericarditis**											
Brucato et al. (2016)	21	ANK at 2 mg/kg per day, up to 100 mg	recurrence of pericarditis and time to recurrence after randomization (median time to flare)	time to response in the open-label phase (time frame, 60 days), and percentage of patients with corticosteroid withdrawal at 6 weeks	18.2%	90%	All patients had a complete response to open-label ANK treatment at day 8, All patients successfully discontinued corticosteroids within 6 weeks and proceeded to the double-blind withdrawal phase.		20	5.8/10 at day 0 and 1.6/10 at day 60 (10 resembles the worst QoL)	0 vs. 0

**Rheumatoid arthritis (RA)**											
Source	Patients treated	Verum	Primary endpoint (1°) investigated	Secondary endpoint (2°) investigated	Verum reaching 1°	Control reaching 1°	Verum reaching 2°	Control reaching 2°	Adverse events (AEs) verum	QoL	Serious adverse events (SAEs)
Niu et al. (2011)	50	ANK 80 mg	ACR response rate after 4,8,12,16,20 and 24 weeks	no clinical secondary endpoint	52.6 % reached ACR20 after 4 weeks, 66.7% after 24 weeks. 5.3% reached ACR70 after 4 weeks, 36.1% after 24 weeks.	25% reachted ACR20 after 4 weeks, 55.4 after 24 weeks. 8.3% reached ACR70 after 4 weeks and 9.1% after 24 weeks.	no clinical secondary endpoint	no clinical secondary endpoint	2	no information	3
Bao et al. (2011)	54	ANK 80 mg	ACR20 at 24 weeks	ACR50 and ACR70 responses at 24 weeks, and DAS28	64%	17%	38% for ACR50, 17% for ACR70, the decrease of DAS28 was greater thanin placebo	0% for ACR50 and ACR70.	no information	no information	no information
Fleischmann et al. (2006) extension trial from 2003	1399	ANK 100 mg	rates of all AEs	no secondary endpoint	96%	no information	no secondary endpoint	no secondary endpoint	1'295	no information	616, infections, disease progression, fractures, and pneumonia
Bresnihan et al. (2004) extension trial from 1998	472	ANK 30 mg, 75 mg, or 150 mg/day	proportion of subjects who attained an ACR20 response at week 48	The 48-week change from baseline in radiographic progression	At 48 weeks, 44%, 53%, and 49% of the patients who continued to receive ANK 30, 75, and 150 mg/day ANK showed	Of the patients who had received placebo and were randomized to receive ANK 30 (n = 30), 75 (n = 24), and 150	erosion score of patients who received ANK treatment for 48 weeks was 1.15, which was significantly less than 2.03	erosion score 48 weeks: 2.03 (p = 0.006)	103	no information	no information

					ACR 20 responses	mg/day (n = 22) at 24 weeks, 50%, 44%, and 71%, respectively, had ACR 20 responses at 48 weeks	observed in the patients originally randomized to placebo (p = 0.006)				
Genovese et al. (2004)	242	ANK 100 mg	proportion of patients achieving an ACR 50% (ACR50) response (16) at week 24	EULAR response at week 24	31%	41%	EULAR response at week 24 73%	EULAR response at week 24 79%	153	no information	12 vs. 2 (pneumonia, gastroenteritis, zoster, lymphoma, neuralgia, back pain, chest pain, TIA, pyelonephritis, dyspnea, gastric ulcer, personality disorder, pneumonitis)
Tesser et al. (2004)	1399	ANK 100 mg	incidence of infections, particularly upper respiratory infections and serious infections;	AEs; and injection site reactions	14.3%	19.1%	7.7%	7.8%	see secondary endpoint	no information	87 vs. 9
Cohen et al. (2004)	501	ANK 100 mg	ACR20 response at week	change from baseline at week 24 in individual ACR components	38%	22%	ACR50 (17%), ACR70 (6%)	ACR50 (8%), ACR70 (2%)	225	no information	10 vs. 8
Fleischmann et al. (2003)	1399	ANK 100 mg	AEs (including infections), discontinuation from study	no secondary endpoint	92%	92.2%	no secondary endpoint	no secondary endpoint	1'027	no information	87 vs. 9 (worsening of RA, respiratory, infections,

			due to AEs, and death								cellulitis, bone infections)
Cohen et al. (2003)	9	ANK, 0.04, 0.1, 0.4, 1, or 2 mg/kg once daily for 24 weeks	change in HAQ-DI from baseline to week 24	no secondary endpoint	HAQ-DI was statistically significant and positively related to the dose of ANK (p = 0.0014) with doses of 1mg or 2 mg/kg	no control	no secondary endpoint	no secondary endpoint	no control	no information	no information
Nuki et al. (2002)	472	ANK 30 mg, 75 mg, or 150 mg/day	proportion of subjects who attained an ACR20 response at week 48	change from baseline at week 48 in individual ACR components	all doses: 46%	51%	ACR50 18% ACR70 3%	ACR50 21% ACR70 3%	injection site reactions 282, 2%	no information	no information
Scott et al. (2016) (CARDERA-2)	154	ANK 100 mg daily and MTX started at 7.5 mg/week, and increased two weekly by 2.5 mg to 15 mg/week	Erosive progression, as captured by changes from baseline in modified Larsen scores	Changes from baseline in DAS28, HAQ and EQ-5D scores alongside ACR-20, 50 and 70 responder rates	mean change from baseline of 2.50 and 5.10	mean change from baseline of 4.16 and 5.20	At 12-months more patients attained an ACR20 and ACR50 response with ANK-MTX	Greater reduction of DAS28, HAQ score, improvement of EQ-5D score at 12 and 24-months with MTX monotherapy, At 24-months more patients attained ACR20, ACR50 and ACR70 responses with MTX monotherapy	70	No benefit in QoL	17
Cohen et al. (2002)	419	ANK 0.04, 0.1, 0.4, 1.0, or 2.0 mg/kg every day	ACR20 response at week 12	ACR20 response at week 24, ACR50 and	0.04mg 25%, 0.1mg 35%, 0.4mg 25%,	19%	only significant was ACR20 1mg 42%,	ACR20 23%, sustained ACR20 15%	injection site reaction 0.04mg 19%, 0.1mg 38%,	no information	no information

				ACR70 responses at weeks 12 and 24, sustained ACR20 response, and change from baseline at weeks 12 and 24 in ACR improvement criteria components, ESR, and duration of morning stiffness.	1mg 46%, 2 mg 38%		sustained ACR20 response 0.04mg 13%, 0.1mg 30%, 0.4mg 22%, 1mg 31%, 2 mg 35%,		0.4mg 56%, 1mg 64%, 2 mg 63%, headache 14-34%, upper respiratory tract infections 15-22%		
Bresnihan et al. (1998)	472	ANK 30 mg, 75 mg, or 150 mg/day	clinical response with ACR composite score	number of swolle, painful joints, patients and investors assessment of disease activity, patients assessment of pain, duration of morning stiffness, healt assessment questionnaire score, ESR, radiogrraph Larson score	30 mg 39%, 75 mg 34%, 150 mg 43%	27%	no information	no information	30 mg 50%, 75 mg 73%, 150 mg 81%	no information	27 vs. 7

Schnitzler's syndrome											
Source	Patients treated	Verum	Primary endpoint (1°) investigated	Secondary endpoint (2°) investigated	Verum reaching 1°	Control reaching 1°	Verum reaching 2°	Control reaching 2°	Adverse events (AEs) verum	QoL	Serious adverse events (SAEs)
Wendling et al. (2012)	6	ANK 100 mg daily	clinical response	reduction of pain VAS, patients disease activity assessment VAS, BASDAI in cases of axial disease,	83.3%	no control	disease activity VAS from 7 to 3.25 after 1 month and 2.5 after 5 months, general reduction of BASDAI	no control	2	no information	none
**SAPHO**											
Rowczenio et al. (2018)	21	ANK, dosing unclear	treatment response with Quality Metric SF36v2 Health Survey	no secondary endpoint	90.4%	no control	no secondary endpoints	no control	only minor	Significant improvement of the mean QOL scores in each domain	0
Gran et al. (2010)	3	ANK 100 mg	treatment response	no secondary endpoint	100%	no control	no secondary endpoints	no control	no information	no information	no information
de Koning (2006)	3	ANK 100 mg	treatment response	no secondary endpoint	100%	no control	no secondary endpoints	no control	no information	no information	no information
**Sjögren's syndrome**											
Norheim el al (2012)	26	ANK 100 mg	group-wise comparison of fatigue scores at week 4	change in fatigue scores within each treatment group during the study, and safety and tolerability of ANK in pSS.	-30%	-10%	There were no significant changes in FSS scores in either treatment group during the study Fatigue levels returned to baseline one week after the last injection in both groups (week 5)		2	no information	2 vs. 2

Systemic juvenile idiopathic arthritis (sJIA)											
Source	Patients treated	Verum	Primary endpoint (1°) investigated	Secondary endpoint (2°) investigated	Verum reaching 1°	Control reaching 1°	Verum reaching 2°	Control reaching 2°	Adverse events (AEs) verum	QoL	Serious adverse events (SAEs)
Quartier et al. (2011)	24	ANK 2 mg/kg, max. 100 mg	Number of patients fulfilling the three following conditions: (1) ACRpedi 30 response; (2) absence of disease-related fever (body temperature <38°C over the past 8 days) and (3) 50% decrease compared with D1 or normalisation of both CRP and ESR values	Modified ACRpedi 30, 50, 70 and 100 responses	67%	8%	92, 58, 42, 0 (in %)	58, 0, 0, 0 (in %)	14	No information	0 vs. 0
Ilowite et al. (2009)	11	ANK 1 mg/kg daily, up to a maximum daily administratio n of 100 mg/day	incidence of treatment-emergent AEs	proportion of patients with response and disease flares in the 16-week blinded phase, time to disease flare and changes in the JRA core components at week 28,	72%	68%	73% in open label were responders, 22% disease flare, significant longer time to flare, (P = 0.057) , significant improvement of JRA core components,	50% disease flare	17 (68%)	no information	3 (nephrosis, viral infection, hepatitis)

							CHAQ, and ESR at 28 weeks				
**TNF-receptor-1 associated periodic syndromes (TRAPS)**											
Source	Patients treated	Verum	Primary endpoint (1°) investigated	Secondary endpoint (2°) investigated	Verum reaching 1°	Control reaching 1°	Verum reaching 2°	Control reaching 2°	Adverse events (AEs) verum	QoL	Serious adverse events (SAEs)
Gattorno et al. (2008)	5	ANK 1.5 mg/kg/day	treatment response	no defined	100%	no control	no control	no control	only minor (skin rash)	no information	none
**Type 1 diabetes mellitus (T1DM)**											
Moran et al. (2013)	69	ANK 100 mg daily	comparison of AUC of stimulated C-peptide response over a 2-h MMTT at the 9 month visit	peak and time to peak of MMTT stimulated C peptide, fasting glucose concentration, HbA1c and insulin dose over time	The difference between groups was not significant	no information	ANK did not affect incremental or peak C-peptide response to a MMTT, percentage of HbA1c or fasting and AUC glucose concentration during an MMTT, plasma IL-6 or overall CRP concentrations, or time to peak C-peptide response to an MMTT. No participant achieved an insulin-free state with maintenance of an HbA1c percentage less than 7·5%.		90	no information	0

**BERMEKIMAB (BER)**											
**HS**											
Source	Patients treated	Verum	Primary endpoint (1°) investigated	Secondary endpoint (2°) investigated	Verum reaching 1°	Control reaching 1°	Verum reaching 2°	Control reaching 2°	Adverse events (AEs) verum	QoL	Serious adverse events (SAEs)
Kanni et al. (2018)	20	7.5 mg/kg of MABp1/BER every two weeks for 12 weeks	positive HiSCR at week 12	HiSCR at week 24, decrease of at least two assessed scores PGA, DAS Sartorius score, visual analog scale for pain, and DLQI median Time to HS exacerbation, changes in skin ultrasonography variables	60%	10%	HiSCR at week 24 6, time to HS exacerbation 11 weeks, decrease of at least two of the assessed scores 80%, decrease of total lesion depth 77.8%	HiSCR at week 24 0, time to HS exacerbation 7 weeks, , decrease of at least two of the assessed scores 40%, decrease of total lesion depth 22%	19 cases (HS exacerbation)	improved QoL in 80% treated with BER compared to 40% in placebo	2 vs. 2 all of them HS exacerbations that required hospitalisation

**CANAKINUMAB**											
**AOSD**											
Source	Patients treated	Verum	Primary endpoint (1°) investigated	Secondary endpoint (2°) investigated	Verum reaching 1°	Control reaching 1°	Verum reaching 2°	Control reaching 2°	Adverse events (AEs) verum	QoL	Serious adverse events (SAEs)
Kedor et al. (2020)	36	CAN 4mg/kg, max 300 mg every 4 weeks	change in DAS (ΔDAS28(ESR)>1.2) at week 12	DAS28 (ESR and CRP), fever episodes, physician assessment of disease activity, limitation of motion (LOM), (HAQ)–Disability Index QoL, SF-36, ACR, EULAR response, ACR30 at week 4, 8 and 12	66.7%	41.2%	Although the response rates for DAS28 (ESR and CRP), fever episodes, physician assessment of disease activity, limitation of motion (LOM), HAQ, ACR30 and EULAR response were higher in the verum group, the differences compared with the placebo group did not achieve statistical significance		17	no significant change	4 SAE in verum group, hepato-toxicity, patella-femoral pain syndrome, hypotonia, deep vein thrombosis; 5 SAE in placebo, abdominal pain, cholecystitis, fracture, hand fracture, medical device removal
**BD**											
Vitale et al. (2014)	3	CAN 150 mg every 6 weeks	complete response	no secondary endpoint	100%	no secondary endpoint	no secondary endpoint	no secondary endpoint	0%	no information	
**CAPS/MWS**											
Lachmann et al. (2009)	31	CAN 150 mg every 8 weeks	proportion of patients with a relapse of CAPS during CAN treatment, as compared	proportion of patients with a complete response in part 1, values of inflammatory	0%	81%	97%, 0%, mean CRP 1, SAA 6.1	97%, 81%, mean CRP 19.9 and SAA 43.3	15	no information	2 SAE (lower urinary tract infection and sepsis, and increased intraocular pressure with

			with placebo, in part 2.	markers (mean increase CRP in part 2, median SAA)							unilateral blindness)
Koné-Paut et al. (2011)	31	CAN 150 mg every 8 weeks	complete response week 8, maintained complete response at week 24, resolution of symptoms at end of part 3	HRQoL	80%, 85%, 96.8%	80%, 25%, 96.8%	improvement of FACIT-F by 13.5, SF36 PCS by 9.5 and SF-36 MCS by 3.6 points after 8 weeks	No information	15	see secondary endpoint	2 SAE (lower urinary tract infection and sepsis, and increased intraocular pressure with unilateral blindness)
**FMF**											
Benedetti et al. (2018)	63	CAN 150 mg, or 2 mg per kilogram of body weight (for patients weighing ≤40 kg) every 4 weeks	Number of patients with complete response, defined as resolution of the baseline flare at day 15 and no new flare until week 16.	proportion of patients who had a PGA score of less than 2, a CRP level of 10 mg or less per liter, or a SAA level of 10 mg or less per liter at week 16, in epoch 3, the proportion of patients receiving who had no flare	61%	6%	PGA score of less than 2 65%, a CRP level of 10 mg or less per liter 68%, a SAA level of 10 mg or less per liter 26%, proportion of patients without flare 77.8%	PGA score of less than 2 9%, a CRP level of 10 mg or less per liter 6%, a SAA level of 10 mg or less per liter 0%, proportion of patients without flare 30%	1.34 per 100 patient years	no information	3 vs. 7
Ozen et al. (2020)	60	CAN 150 mg q8w to 150 mg q4w to 300 mg q4w, under 40kg 2 or 4mg/kg	complete response, defined as resolution of the baseline flare at day 15 and no new flare	number of flares per patient, the PGA of disease activity and the analysis of CRP and	see study 2018	no control	no flares (35/60, 58%) or a single flare (23/60, 38%), incidence of flares was similar in the two cumulative dose groups, Low PGA scores were maintained, low levels of CRP were generally maintained for 72 weeks, with		1.53 per 100 patient days,	no information	23 SAE (neutropenia, PG, sinusitis, cellulitis, gastroenteritis, infectious colitis, peritonitis,

			until week 16.	SAA serum for 72 weeks			median CRP concentrations lower than 10 mg for all measurements between week 41 and week 113, Median SAA levels observed remained over the 10 mg/L limit of normal, but under the 30 mg/L threshold				urinary tract infection)
**Gout**											
Source	Patients treated	Verum	Primary endpoint (1°) investigated	Secondary endpoint (2°) investigated	Verum reaching 1°	Control reaching 1°	Verum reaching 2°	Control reaching 2°	Adverse events (AEs) verum	QoL	Serious adverse events (SAEs)
Schlesinger (2011)	391	CAN single dose of 25 mg, 50 mg, 100 mg, 200 mg, or 300 mg on day 1, or four CAN doses administered at 4-weekly intervals (50 mg on day 1 and at week 4, and 25 mg at weeks 8 and 12)	determination of the CAN dose producing equivalent efficacy to that achieved with COL 0.5 mg, with respect to the mean number of flares per patient occurring within 16 weeks post randomisatio n	mean number of flares per patient, proportion of patients with at least one flare, time to first flare, average duration of flares and CRP levels	The estimated CAN dose with equivalent efficacy to COL was below the range of CAN doses tested		62% to 72% reduction in the mean number of flares per patient for CAN, doses ≥50 mg versus COL; percentage of flares 15% to 27%; 64% to 72% reduction in the risk of experiencing at least one flare; duration of flares 2.8–4.6 days, For all CAN doses ≥50 mg, median CRP values remained consistently lower than in COL	percentages of flares 44%, duration of flares 5.1 days	54%	no information	4 vs. 1

Schlesinger (2011)	191	single dose of CAN at 1 of 5 doses (10, 25, 50, 90, or 150 mg)	determination of the CAN dose that produced equivalent efficacy to that achieved with TA 40 mg 72 hours after treatment, according to the patient's assessment of pain on a VAS.	time to 50% reduction in pain, time to recurrence of flare, reductions in CRP and SAA levels, use of rescue medication, and physician's and PGA of response to treatment, QoL	all CAN doses		reduction from baseline in pain intensity -11.5-19.2 mm, relative risk reduction 94% for flare with CAN 150 mg versus TA, mean SF-36 physical component summary score increased by 12.0 points from baseline to 48.3 at seven days, post-dose; responses to the CAN 150 mg dose were superior to responses to TA 40 mg in all secondary endpoints,	41%	Improvements of all aspects of QoL questionnaires, see secondary endpoints	4 vs. 1
Schlesinger (2012) β-RELIEVED and β-RELIEVED-II trial	456	CAN 150 mg, minimum period between two consecutive study drug administrations was 14 days	pain intensity (VAS score) in the most affected joint at 72 h postdose and time to first new flare over the first 12 weeks	time to first new flare over the entire 24 weeks, global assessments by patients and physicians (each evaluated on a 5-point Likert scale); assessment of joint tenderness (evaluated on 4-point scale), swelling (evaluated on a 3-point scale) and erythema (evaluated on a 3-point scale) by	25mm, difference in pain score between treatments was −10.7 mm, mean number of new flares per patient was significantly lower with CAN (0.19 vs. 0.51)	35mm	CAN delayed the time to first new flare by 62%, at least one new flare during the core study (16% vs. 35.8%) significantly better responses to treatment versus TA according to PGA (OR, 2.2; p≤0.0001) and physician global assessment (OR, 2.3; p≤0.0001), and significantly less tenderness (OR, 2.2; p≤0.0001), swelling (OR, 1.7; p≤0.01), and erythema (OR, 0.6; p≤0.05), CAN produced rapid decreases in median levels of CRP and SAA; levels were consistently suppressed over the entire 24-week period and were lower at each time point in the CAN group	66.2%	no information	13 vs. 10 (angina pectoris; arrhythmia; myocardial ischemia and renal artery occlusion; glaucoma; gastritis and chronic renal failure; spondylolisthesis, spinal cord ischemia and lumbar spinal stenosis; jaw abscess; increased prostate-specific antigen; hyperglycaemia; device dislocation;

				physicians; values of infl ammatory markers; and safety and tolerability						and pneumonia)
So et al. (2010)	191	single dose of CAN at 1 of 5 doses (10, 25, 50, 90, or 150 mg)	determination of the CAN dose that produced equivalent efficacy to that achieved with TA 40 mg 72 hours after treatment, according to the patient's assessment of pain on a VAS.	time to 50% reduction in pain, time to recurrence of flare, reductions in CRP and SAA levels, use of rescue medication, and physician's and PGA of response to treatment	all used CAN doses		reduction from baseline in pain intensity -11.5-19.2 mm, relative risk reduction 94% for flare with CAN 150 mg versus TA, responses to the CAN 150 mg dose were superior to responses to TA 40 mg in all secondary endpoints, improvement of QoL was seen in all dose groups	41%	no information	4 vs. 1

HIDS											
Source	Patients treated	Verum	Primary endpoint (1°) investigated	Secondary endpoint (2°) investigated	Verum reaching 1°	Control reaching 1°	Verum reaching 2°	Control reaching 2°	Adverse events (AEs) verum	QoL	Serious adverse events (SAEs)
Benedetti et al. (2018)	72	CAN 150 mg, or 2 mg per kilogram of body weight (for patients weighing ≤40 kg) every 4 weeks	complete response, defined as resolution of the baseline flare at day 15 (PGA score of <2 plus CRP level of ≤10 mg per liter or a reduction by ≥70% from baseline) and no new flare (PGA score of ≥2 and CRP level of ≥30 mg per liter) until week 16.	proportion of patients who had a PGA score of less than 2, a CRP level of 10 mg or less per liter, or a SAA level of 10 mg or less per liter at week 16 and, in epoch 3, the proportion of patients receiving CAN or placebo every 8 weeks who had no flare	35%	5.7%	PGA score of less than 2 46%, a CRP level of 10 mg or less per liter 41%, a SAA level of 10 mg or less per liter 14%, proportion of patients without flare 50%	PGA score of less than 2 6%, a CRP level of 10 mg or less per liter 6%, a SAA level of 10 mg or less per liter 3%, proportion of patients without flare14.3%	251 per 100 patient years	no information	3 vs. 11
**PG**											
Kolios et al. (2018)	5	CAN 150-300 mg per month depending on GPA until week 8, then stop	clinical improvement (PGA at least-1 from baseline), complete remission	DLQI, percentage of patients with complete clinical remission and the percentage of patients with partial clinical improvement	80%	no control	60% showed complete healing, 4 patients showed a reduction in DLQI, diameter from 4 32 cm at visit 1 to 0.73 cm at visit 7	no control	2	4 patients showed a significant reduction in DLQI	1 (scrotal ulcers)

				at weeks 2, 4, 8 and 12; the change in target-lesion diameter and area compared with baseline							
**RA**											
Source	Patients treated	Verum	Primary endpoint (1°) investigated	Secondary endpoint (2°) investigated	Verum reaching 1°	Control reaching 1°	Verum reaching 2°	Control reaching 2°	Adverse events (AEs) verum	QoL	Serious adverse events (SAEs)
Alten et al. (2011)	274	CAN 150 mg SC every 4 weeks (q4wk), CAN 300 mg SC (2 injections of 150 mg SC) every 2 weeks, a 600 mg IV loading dose of CAN followed by 300 mg SC every 2 weeks', or placebo SC every 2 weeks	ACR 50 criteria at 12 weeks of the 150 mg every 4 weeks group in comparison to placebo	ACR 20 and ACR 70 at week 12; ACR 20, ACR 50, and ACR 70 responses at any visit; ACR component variables; SF-36; FACIT-F; DAS28, EULAR and HAQ	26.5%	11.4%	percentages of ACR 70 responders were higher in the CAN 150 mg SC q4wk group than in the placebo group except at week 2. Percentages of ACR 70 responders in the CAN 300 mg SC q2wk group were higher than in the placebo group at all visits. Significant differences favoring CAN 150 mg SC q4wk vs. placebo DAS28, PGA ($p < 0.05$ for all comparisons), as well as the HAQ and DAS28 scores ($p < 0.05$ for both comparisons). EULAR criteria was likewise higher with CAN 150 mg SC q4wk (25%) than with 300 mg SC q2wk (18.8%), 600 mg IV loading dose plus 300 mg SC q2wk (12.7%), or placebo (18.6%). greater average decreases in ESR levels compared to baseline were observed in the three CAN treatment groups than in placebo		52% in all CAN, CAN 150 mg every 4 weeks 46.4%, compared to placebo 53%,	significant improvements in HAQ, SF-36 and FACIT-F scores in all CAN groups compared to placebo	8 vs. 5
**Schnitzler's syndrome**											

Source	Patients treated	Verum	Primary endpoint (1°) investigated	Secondary endpoint (2°) investigated	Verum reaching 1°	Control reaching 1°	Verum reaching 2°	Control reaching 2°	Adverse events (AEs) verum	QoL	Serious adverse events (SAEs)
Krause et al. (2017)	20	CAN 150 mg single dose,	complete clinical responders at day 7	changes PGA and patient-reported Schnitzler activity score assessments of disease activity, changes in CRP and SAA, and changes in DLQI and SF-36	71%	0%	PGA -11, CRP -8, SAA -389, DLQI -5	PGA 0, CRP -0, SAA -13, DLQI 0.5	0	DLQI -10 vs. 0.5	0
**sJIA**											
Ruperto et al. (2018)	144	CAN 4mg/kg, max 300 mg every 4 weeks; CAN dose was tapered in the LTE to 2 mg/kg every 4 weeks in patients who were GC free	at 6 Months and 3 years aJIA-ACR 50/70/90; clinically inactive disease (CID)/clinical remission on medication (CR), Juvenile Arthritis DAS (JADAS Score) with high or low diasease activity	discontinuation of GCS at 6 months, 2 and 5 years	6 months: aJIA-ACR 50/70/90 73.4%, 65.5%, 52%, CID 32.8%; CR 18.6%; 3 years: aJIA-ACR 50/70/90 54.8%, 53.7%, 49.7%, CID 36.7%, Cr 28%;	no control	6 months (29.7%), 2 years (39.8%), 5 years (15.6%)	no control	exposure-adjusted incidence rate of AEs was 796.69/100 patient-years	no information	194 (relapse, macrophage activation snydrome, fever)
Ruperto et al. (2012)	Trial 1: 84; Trial 2: 100	CAN 4mg/kg, max 300 mg every 4 weeks	Trial 1: proportion of patients with an adapted JIA ACR 30	T1 + T2: JIA ACR 50, JIA ACR 70, JIA ACR 90, and JIA ACR 100	T1: 84% T2: no information	T1: 10% T2: no information	T1: 79%, 67%, 47%, 33%, 30%; T2: 84%,	T1+T2: 5%, 2%, 2%, 2%, 0%;	T1: 49; T2: 272	no information	T1: 2 vs. 2; T2: 6 vs. 6

			response ; Trail 2: time to flare	responses and inactive disease; T2: 25% GCS reduction,			82%, 76%, 64%, 62%				
**TRAPS**											
Source	Patients treated	Verum	Primary endpoint (1°) investigated	Secondary endpoint (2°) investigated	Verum reaching 1°	Control reaching 1°	Verum reaching 2°	Control reaching 2°	Adverse events (AEs) verum	QoL	Serious adverse events (SAEs)
Benedetti et al. (2018)	46	CAN 150 mg, or 2 mg per kilogram of body weight (for patients weighing ≤40 kg) every 4 weeks	complete response, defined as resolution of the baseline flare at day 15 (PGA score of <2 plus CRP level of ≤10 mg per liter or a reduction by ≥70% from baseline) and no new flare (PGA score of ≥2 and CRP level of ≥30 mg per liter) until week 16.	proportion of patients who had a PGA score of less than 2, a CRP level of 10 mg or less per liter, or a SAA level of 10 mg or less per liter at week 16 and, in epoch 3, the proportion of patients receiving CAN or placebo every 8 weeks who had no flare	45.4%	8.3%	PGA 45%, CRP 36%, SAA 27%, no flare 75%	PGA 4%, CRP 8%, SAA 0%, no flare 40%	112 per 100 patient-years	no information	3 vs. 3
**T1DM**											
Moran et al. (2013)	69	CAN 2 mg/kg (maximum 300 mg) monthly	comparison of the area under the curve (AUC) of stimulated C-peptide response over a 2-h MMTT	C-peptide slope over time, the time in trial until peak C peptide is less than 0·2 nmol/L,	difference between groups was not significant	no information	35% reduction in CAN, 33% reduction in placebo of C-peptide within a year. Time to stimulated peak C peptide less than 0·2 nmol/L, did not differ between the two groups Percentages of HbA1c increased gradually over time		81	no information	2

			at the 12 month visit	HbA1c and insulin dose over time			and were similar between CAN-treated and placebo-treated participants at 1 year (p=0·76). Similarly, there was no difference in insulin dose at 1 year between groups				
**Urticarial vasculitis (UV)**											
Source	Patients treated	Verum	Primary endpoint (1°) investigated	Secondary endpoint (2°) investigated	Verum reaching 1°	Control reaching 1°	Verum reaching 2°	Control reaching 2°	Adverse events (AEs) verum	QoL	Serious adverse events (SAEs)
Krause et al. (2013)	10	CAN 300 mg single dose	change in mean UV activity score (UVAS)	changes in the physician and PGA of disease activity, changes in inflammation markers CRP and ESR	60%	no control	disease activits mean improvement of 41%, with 4 of 10 patients demonstrating a >50% improvement) compared with day 0, complete clinical response in 20%, 40% improvement ind QoL	no control	9%	improvement of 40% in QoL	0

GEVOKIZUMAB (GEV)

Source	Patients treated	Verum	Primary endpoint (1°) investigated	Secondary endpoint (2°) investigated	Verum reaching 1°	Control reaching 1°	Verum reaching 2°	Control reaching 2°	Adverse events (AEs) verum	QoL	Serious adverse events (SAEs)
**BD**											
Tugal-Tutkun et al. (2018)	83	GEV 60 mg every 4 weeks	time to first acute ocular exacerbation	number of patients with acute occular exacerbations , changes in visual acuity, occular scores, GCS dose, safety	did not significantly impact the time to first ocular exacerbation		no of patients with exacerbation 35%, 92% patients receiving GCS <10 mg PDN at recurrence	no of patients wit exacerbation 34.9%, 80% patients receiving GCS <10 mg PDN at recurrence	92.7% 311 events	no information	13 (31.7%) vs. 14 (32.6%) no specific pattern
**T1DM**											
Seelig et al. (2016)	26	GEV 0.3 mg/kg every 4 weeks for 4 months	change in the area under the concentration –time curve (AUC) for C-peptide during the MMT between baseline and after 4 months of treatment	changes in levels of 2-h AUC C-peptide, HbA1c, fasting glucose, fasting glucagonand cortisol, CRP and lipids, and GAD65and IA-2 autoantibodies	there was no significant differenceat 4 and 12 months in 2-h AUC C-peptide levels		fasting glucose levels and HbA1clevels remained similar in both groups compared with base-line (Table 1). Also, fasting glucagon, cortisol, high-sensitivityCRP, lipids, GAD65 and IA-2 autoantibodies remained similar compared to baseline		100% 16 AE	no information	1 vs. 0 CMV infection

RILONACEPT (RIL)

Source	Patients treated	Verum	Primary endpoint (1°) investigated	Secondary endpoint (2°) investigated	Verum reaching 1°	Control reaching 1°	Verum reaching 2°	Control reaching 2°	Adverse events (AEs) verum	QoL	Serious adverse events (SAEs)
**AOSD**											
Petryna et al. (2012)	3	RIL 220 mg loading dose and 160 mg weekly	clinical response (fever, rash, involved joints)	laboratory response (wbc, ferritin, CRP, ESP, PDN reduction)	100%	no control	100%	no control	no information	no information	no information
**CAPS/MWS**											
Hoffman et al. (2008) same publication but two consecutive studies	47	RIL loading dose 320 mg followed by 160 mg weekly	mean change in the mean key symptom score	number of multisympto m disease flare days, number of single-symptom disease flare days, maximum severity of any symptom, mean change in patients and PGA of disease activity, CRP and SAA changes,	84%	13%	at least 30% of symptom reduction in 96%, at least 50% reduction in 87%, 75% reduction in 70%	at least 30% of symptom reduction in 29%, at least a 50% reduction in 8%, 75% reduction 0%	74% (17 AEs)	see secondary endpoint	0 vs. 0
Hoffman et al. (2008) same publication	45	RIL 160 mg, 9 weeks of weekly single-blind	mean change in the mean key symptom score	number of multisympto m disease flare days,	significantly maintaining low mean key point score in comparison to placebo		significant maintaining the low number of multisymptom and single-symptom disease flare days, maximum score		68% (15 AEs)	Significant improvement of QoL	0 vs. 0

but two consecutive studies		treatment with SCj, followed by 9 weeks 160 mg weekly		number of single-symptom disease flare days, maximum severity of any symptom, mean change in patients and PGA of disease activity, CRP and SAA changes,			for any single symptom, mean scores fore each 5 key symptoms, improvement of physicians and PGA of disease activity, limitations in patients daily activities, and levels of CRP and SAA			compared to placebo	
**Deficiency of interleukin-1 receptor antagonist (DIRA)**											
Source	Patients treated	Verum	Primary endpoint (1°) investigated	Secondary endpoint (2°) investigated	Verum reaching 1°	Control reaching 1°	Verum reaching 2°	Control reaching 2°	Adverse events (AEs) verum	QoL	Serious adverse events (SAEs)
Carg et al. (2017)	6	RIL loading dose 4.4mg/kg,followed by 2.2 mg/kg weekly, dose escalation 4.4mg/kg weekly if needed	maintain/achieve remission at 6, 12 and 24 months	patients questionnaire (CHAQ, PesQL), diary scores, acute phase reactants, growth and weight, changes in bone mineral density,	100%, 5 out of 6 needed dose escalation	no control	improvement in patients questionnaire (CHAQ, PesQL), diary scores, acute phase reactants, growth and weight, even normalization of bone mineral density,	no control	100% (upper respiratory infection, otitis media, rash, pharingytis, GI symptoms, allergic reaction to food and acetaminophen)	see secondary enpoint	0
**FMF**											
Hashkes et al. (2012)	14	RIL 2.2 mg/kg (maximum,160 mg) weekly	difference in the frequency (Attacks per month) of FMF attacks (at least	proportion of treatment courses: with no attacks; decrease in attacks	76%	39%	no attacks 7, decrease in attacs >50% 18, days to first attack 20, days to	no attacks 0, decrease in attacs >50% 8, days to first attack 15, days to	73 AEs	statistically and clinically significant differences between treatment	4 vs. 3

			improvement of 40%)	greater than 50% compared with the baseline rate during screening; time to development of attacks; length of time received treatment; proportion of time in attack; acute-phase reactants, PGA; HRQOL.			second attack >90, length of attacks (d) 2.8, QOL psy 53 phy 33	second attack 36, length of attacks (d) 3.2, QOL psy 49 phy 20, No statistically significant differences in the proportion of time in attack,, time participants were treated with RIL, PGA, acute-phase reactants		groups in physical but not psycho-social aspects of HRQOL	
**Gout**											
Source	Patients treated	Verum	Primary endpoint (1°) investigated	Secondary endpoint (2°) investigated	Verum reaching 1°	Control reaching 1°	Verum reaching 2°	Control reaching 2°	Adverse events (AEs) verum	QoL	Serious adverse events (SAEs)
Terkeltaub (2013)	225	RIL 320 mg at baseline	change in pain in the index joint from baseline to the average of values at 24, 48 and 72 hours	proportion of patients requiring rescue medication., CRP	-1.6	-1.4	rescue therapy 3% (RIL) and 4.3% (placebo) no significant differences were observed with RIL plus indomethacin relative to indomethacin monotherapy, but indomethacin monotherapy was significantly superior to RIL monotherapy at all time points		46%	no information	3 vs. 0
Schumacher et al. (2012)	83	RIL loading dose 320 mg followed by 160 mg weekly	number of gout flares per patient from day 1 through week 12	proportion of patients with flares, the number of gout flare days per	0.15; 81% decrease in flares with RIL at 12 weeks	0.79	proportions of patients with flares 6, gout flare days per patient 1.41	proportions of patients with flares 19 (45%), gout flare days per patient 5.17,	61%	no information	1 vs. 2

				patient, and the number of days per patient with a pain score of more than 5			(14.6%), days with pain score >5 4.5 0.78,	days with pain score >5 4.5			
Schumacher et al. (2012)	240	RIL 80 mg or 160 mg weekly (double loading dose at day 1)	number of gout flares per patient through week 16	proportion of patients with min 1 flares, the proportion of patients with >2 flares, the mean number of gout flare days, and the mean number of days with a pain severity score >5.	80 mg -73%, 160 mg 80%,	0%	1 gout flare: 80 mg 18.8%, 160 mg 16.3%, multiple gout flares 80 mg 5%, 160 mg 3.7%	1 gout flare 31.6%, multiple gout flares 31%	63%	no information	5 vs. 3
Mitha et al. (2013)	248	RIL 80 mg or 160 mg weekly (double loading dose at day 1)	number of gout flares per patient through week 16	proportion of patients with min 1 flares, the proportion of patients with >2 flares, the mean number of gout flare days, and the mean number of days with a pain severity score >5.	80 mg -71.3%, 160 mg -72.6%,	0%	1 gout flare 80 mg 25%, 160 mg 20.5%; multiple gout flares 80 mg 8.5%, 160 mg 6%	single flare 56.1%, multiple gout flares 32.9%	65%	no information	8 vs. 4 SAEs occurred with a similar frequency across treatment groups, and none was considered by the investigator to be related to study medication
Sundy et al. (2014)	1315	RIL loading dose 320 mg followed by 160 mg weekly	incidence and types of treatment-emergent AEs, including SAEs	mean number of gout flares per patient; the proportion of patients with ≥ 1 and ≥ 2 flares, the	AE 66%, SAE 3.1%	AE 59%, SAE 3.9%	reduction by 70% in the mean number of gout flares, 25.7% with at least 1 flare, 11.7% with at least 2 flares	51.% with at least 1 flare, 34.7% with at least 2 flares	66% headache, arhtralgia, injection site reaction	no information	31 vs. 13, only 3 drug related., 2 in the placebo group (1 death and 1 case of

				number of flare days, and time to first flare.							cellulitis) and 1 with RIL (drug eruption)
**Recurrent pericarditis**											
Source	Patients treated	Verum	Primary endpoint (1°) investigated	Secondary endpoint (2°) investigated	Verum reaching 1°	Control reaching 1°	Verum reaching 2°	Control reaching 2°	Adverse events (AEs) verum	QoL	Serious adverse events (SAEs)
Klein et al. (2020)	86	RIL loading dose 320 mg (or 4.4mg/kg) followed by 160 mg (or 2.2 mg/kg) weekly for 12 weeks, after that again weekly 160 mg	median time to pericarditis recurrence	withdrawal period: percentage of patient with persisting response at week 16, percentage of days with no pain, percentage of patients with absent symptoms, severety rating scale, run-in period: time to pain response, time to normalization of CRP, time to prespecified treatment response, time by patient discontinues stadard therapy	not able to calculate because there were to few events	8.6 weeks	run-in: time to pain response after first injection (5 days), median time to normalization of CRP 7 days, median time to RIL monotherapy 7.9 weeks; withdrawal: persistant clinical response 17 patients; days with no pain 97%, minimal symptoms in 81% patients	withdrawal: persistant clinical response 4 patients; days with no pain 45%, minimal symptoms in 25% patients	80% (24 AEs)	no information	3 vs. 3 stroke, palpitations, SCC, pyrexia, ileus
**Schnitzler's syndrome**											

Krause et al. (2016)	8	RIL loading dose 320 mg followed by 160 mg weekly	safety and tolerability	change in patient (SchAS activity Score)and physician reported outcomes from baseline, changes in inflammatory markers	no information	no control	50% showed rapid and complete response,	no control	13 AEs	no information	0
**sJIA**											
Source	Patients treated	Verum	Primary endpoint (1°) investigated	Secondary endpoint (2°) investigated	Verum reaching 1°	Control reaching 1°	Verum reaching 2°	Control reaching 2°	Adverse events (AEs) verum	QoL	Serious adverse events (SAEs)
Lovell et al. (2013)	24	RRIL 2.2 mg/kg or 4.4 mg/kg, on days 0, 3, 7, 14, and 21	safety (AE at week 4)	efficacy (ACR 30, 50 an d70, absence of fever and rash), PGA of disease activits (PGA), childhood healt assessment questionnaire, PDN dose	6%	0%	adapted ACR Pediatric 30, 50, and 70 responder rates 57.1%, 57.1%, and 14.3%, respectively, ACR 70 at the 6- and 12-months were 42.9% (3 of 7) and 85.7%. reduced GCS 10 to 3 mg among the 12 patients still receiving PDN at the 12-month, all 10 patients who were still receiving RIL at the end of	in open label no control	23%	no information	3

							the study had discontinued PDN therapy by 24				
Ilowite et al. (2014)	71	RIL loading dose (4.4mg/kg, maximum dose 320 mg) day 0 followed by weekly maintenance doses	time to response during the 12 week efficacy (ACR 30, absence of fever, redukction of GCS at least by 10%)	response rate at week 4, JIA ACR30, 50, 70, inactive disease 23, presence of fever, serositis, symptomatic anemia, abnormal liver function, rash, MAS, incomplete MAS, corticosteroid dose, CHAQ, and PedsQL Generic Core Modules	77%	59%	response rate week 4: 20, ACR 30: 26, ACR50: 21, ACR70: 14; corticosteroid dose decreased more in the RIL arm than in the placebo arm during the efficacy period; response rate week 4: 57%, ACR 30: 74%, ACR50: 60%, ACR70: 40%	response rate week 4: 9, ACR 30: 13, ACR50: 10, ACR70: 4; response rate week 4: 27%, ACR 30: 39%, ACR50: 30%, ACR70: 12%	28%	no information	4 vs. 2, 4 in RIL (relapse, liver function test abnormal, pyrexia, varicella) 2 relapses in placebo, LTE (MAS, relapse, pericarditis, streptococcal phayryngitis)
**T1DM**											
Source	Patients treated	Verum	Primary endpoint (1°) investigated	Secondary endpoint (2°) investigated	Verum reaching 1°	Control reaching 1°	Verum reaching 2°	Control reaching 2°	Adverse events (AEs) verum	QoL	Serious adverse events (SAEs)
White et al. (2018)	13	RIL loading dose 320 mg (or 4.4mg/kg) followed by 160 mg (or 2.2 mg/kg) weekly for 26 weeks	incidence of all cause infections as the primary end-point	incidence of other AEs, hemoglobin A1c (HbA1c), total daily insulin dose, insulin dose-adjusted HbA1c	85 AE	no control	no significant superiority, incidence of other AEs, hemoglobin A1c (HbA1c) increased from 6.8 to 7.3, no significant change in in insuline dose	no control	85 AEs	no information	0

Abbreviations: ACR, American College of Rheumatology; AE, adverse event; ANK, Anakinra; AOSD, adult-onset Still's disease; AZA, azathioprine; BER, Bermekimab; BD, Behcet's disease; CAN, Canakinumab; CAPS, cryopyrin-associated periodic syndrome; COL, colchicine; CRP, C-reactive protein; CSA, cyclosporine A; CYC, cyclophosphamide; DAS, disease activity score; DAS28, 28-joint disease activity score; DIRA, deficiency of interleukin-1 receptor antagonist; DLQI, dermatology life quality index; DMARD, disease-modifying antirheumatic drug; eGFR, estimated glomerular filtration rate; EMA, European Medicines Agency; ESR, erythrocyte sedimentation rate; EULAR, European League Against Rheumatism; FDA, U.S. Food and Drug Administration; Fc, m crystallizable; FMF, familial Mediterranean fever; GC, g; GEV, Gevokizumab; GvHD, graft-versus-host disease, HAQ, health assessment questionnaire; HbA1c, hemoglobin A1c; HIDS, hyper-IgD syndrome; HiSCR, hidradenitis suppurativa clinical response; HS, hidradenitis suppurativa; IGA, investigator's global assessment; IL, interleukin; IV, intravenous(ly); LEF, leflunomide; mAb, monoclonal antibody; MKD, mevalonate kinase deficiency; MTX, methotrexate; MWS, Muckle-Wells syndrome; NSAID non-steroidal anti-inflammatory drug; PDN, prednisone; PG, pyoderma gangrenosum; PGA, patient global assessment; PsARC, psoriatic arthritis response criteria; QoL, quality of life; RA, rheumatoid arthritis; RCT, randomized controlled trial; RIL, Rilonacept; SAA, serum amyloid A; SAE, serious adverse event; SAPHO, ; SAPHO, synovitis, acne, pustulosis, hyperostosis and osteitis ; SC, subcutaneous(ly); SF-36, 36-Item Short Form Survey; sJIA, systemic juvenile idiopathic arthritis; SjS, Sjögren's syndrome; T1DM, type 1 diabetes mellitus; TA, triamcinolone acetonide; TRAPS TNS-receptor-1 associated periodic syndrome; UV, urticarial vasculitis; UVAS, urticarial vasculitis activity score; VAS, visual acuity score
