# Supplementary material for: Systematic Review of Safety and Efficacy of IL-1-Targeted Biologics in Treating Immune-Mediated Disorders
Source: Front Immunol. 2022 Jul 6;13:888392. doi: 10.3389/fimmu.2022.888392 (PMC9296857; doi:10.3389/fimmu.2022.888392)
Supplement: Supplementary file 5 [file Table_5.pdf]

Table S5. Risk of bias.

Study	Bias score
Nordström et al. (18)	20
Grayson et al. (21)	13
Cantarini et al. (22)	6
Kullenberg et al. (25)	12
Lepore et al. (26)	16
Eskola et al. (27)	7
Sibley et al. (28)	19
Ben-Zvi et al. (35)	26
Janssen et al. (39)	23
Antin et al. (49)	21
Tzatanekou et al. (50)	26
Bodar et al. (52)	8
Shakoory et al. (53)	22
Kuemmerle et al. (30)	20
Kuemmerle et al. (29)	17
Jung et al. (54)	14
Beynon et al. (55)	6
Brucato et al. (57)	26
Niu et al. (70)	17
Bao et al. (69)	22
Fleischmann et al. (68)	18
Bresnihan et al. (67)	25
Genovese et al. (66)	25
Tesser et al. (65)	20
Cohen et al. (64)	24
Fleischmann et al. (63)	23
Cohen et al. (62)	23
Nuki et al. (61)	24
Scott et al. (71)	22
Cohen et al. (60)	27
Bresnihan et al. (59)	25
Wendling et al. (79)	12
Rowczenio et al. (73)	6
Gran et al. (74)	5
de Koning et al. (75)	7
Norheim el al (78)	23
Ilowite et al (80)	21
Quartier et al. (81)	26
Gattorno et al. (86)	9

Study	Bias score
Moran et al. (87)	25
Kedor et al(19)	27
Vitale et al. (2823)	6
Lachmann et al. (31)	26
Koné-Paut et al. (32)	24
De Benedetti et al. (37)	26
Ozen et al. (36)	27
Schlesinger, Mysler et al. (43)	27
Schlesinger, Meulemeester et al. (41)	24
Schlesinger (42)	27
So et al. (40)	25
Kolios et al. (56)	12
Alten et al. (72)	26
Krause et al. (76)	21
Ruperto et al. (83)	19
Ruperto et al. (82)	21
Krause et al. (90)	14
Petryna et al. (20)	8
Hoffman et al. (33)	26
Garg et al. (34)	18
Hashkes et al. (38)	23
Terkeltaub (48)	24
Schumacher, Evans et al. (44)	24
Schumacher, Sundy et al. (45)	23
Mitha et al. (46)	25
Sundy et al. (47)	26
Klein et al. (58)	27
Krause et al. (77)	19
Lovell et al. (84)	21
Ilowite et al. (85)	26
White et al. (89)	14
Tugal-Tutkun et al. (24)	24
Seelig et al. (88)	21
Kanni et al. (51)	21

Legend:

Risk of bias

- Low (23-28 points)
- Medium (15-22 points)
- High (0-14 points)
